# Viridicatol from the Deep‐Sea‐Derived Fungus Alleviates Bone Loss by Targeting the Wnt/SHN3 Pathway

**DOI:** 10.1002/advs.202416140

**Published:** 2025-04-07

**Authors:** Chun‐Lan Xie, Shang‐Hui Ye, Yu‐Ting Yue, Bao‐Hong Shi, Jing‐Ping Xu, Lian‐Jie Li, Zheng‐Biao Zou, Matthew B. Greenblatt, Na Li, Xian‐Wen Yang, Ren Xu

**Affiliations:** ^1^ Key Laboratory of Tropical Translational Medicine of Ministry of Education School of Basic Medicine and Life Sciences Hainan Academy of Medical Sciences Hainan Medical University No. 3 Xueyuan Road Haikou Hainan 571199 China; ^2^ Fujian Provincial Key Laboratory of Organ and Tissue Regeneration School of Medicine Xiamen University Xiamen 361102 China; ^3^ The First Affiliated Hospital of Xiamen University‐ICMRS Collaborating Center for Skeletal Stem Cells State Key Laboratory of Cellular Stress Biology Faculty of Medicine and Life Sciences State Key Laboratory of Vaccines for Infectious Diseases Xiang An Biomedicine Laboratory Xiamen University Xiamen 361102 China; ^4^ Department of Pathology and Laboratory Medicine Weill Cornell Medicine New York NY 10065 USA; ^5^ Research Division Hospital for Special Surgery New York NY 10065 USA; ^6^ Shenzhen Key Laboratory of Bone Tissue Repair and Translational Research Department of Orthopaedic Surgery The Seventh Affiliated Hospital of Sun Yat‐sen University Shenzhen 518107 China

**Keywords:** bone loss, bone‐targeting nanovesicles, osteoblasts, Schnurri‐3, viridicatol

## Abstract

As an enticing bone anabolic target, short‐term inhibition of Schnurri‐3 (SHN3) resulted in high‐bone mass due to augmented osteoblast activity. However, no studies are conducted to identify natural products targeting SHN3 inhibition. Herein, a screening strategy for the discovery of marine compounds that facilitate osteoblast differentiation by targeting SHN3 silencing is presented. One leading quinolinone alkaloid, viridicatol (VDC), isolated from deep‐sea‐derived fungus, vigorously promotes osteogenic differentiation via the Wnt/SHN3 signaling pathway in osteoblasts, thereby preventing osteoporosis while enhancing bone‐fracture healing in a mouse model. Subsequently, the SDSSD (Ser, Asp, Ser, Ser, Asp) is further employed to engineer bone‐targeting nanovesicles (BT‐NVs) for the optimal delivery of VDC to osteoblasts, which mitigates the bone loss observed in a severe osteogenesis imperfecta model. Hence, these results initially uncover a promising marine natural product, VDC, targeting the Wnt/SHN3 pathway for the treatment of bone loss and highlighting its translational potential in clinical applications.

## Graphical abstract

Viridicatol (VDC), isolated from the deep‐sea‐derived fungus, enhances osteogenesis in vitro and in vivo by targeting Wnt/SHN3 signaling pathway. It can be delivered via engineered nanovesicles to mitigate bone loss of osteoporosis, fracture repair, and osteogenesis imperfecta.

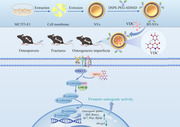

## 1 Introduction

Recent advancements in osteoanabolic therapies have been witnessed, particularly with the advent of the anti‐SOST biologic romosozumab. Nevertheless, there remains a substantial demand for further progress in this domain, as all current anabolic treatments possess considerable limitations.^[^ ^1^ ^]^ For instance, the anti‐sclerostin therapy of romosozumab may be associated with an elevated risk of cardiovascular events, and its anabolic effects diminish after one year of treatment.^[^ ^2^ ^]^ Additionally, anabolic therapies employing parathyroid hormone (PTH) analogs including teriparatide and abaloparatide are confined to a maximum duration of two years on account of discoveries that high doses of PTH were correlated with the development of osteosarcoma in rodent studies.^[^ ^3^ ^]^ Notably, contemporary agents display substantial site‐specific disparities in their osteoanabolic efficacy, with PTH analogues demonstrating significantly greater effectiveness in enhancing bone formation in the spine compared to distal locations like the distal radius.^[^ ^4^ ^]^ Hence, fractures in patients with severe osteoporosis or osteogenesis imperfecta (OI), caused by a decreased strength of bone, require a diverse assortment of mechanistically distinct osteoanabolic therapies.^[^ ^5^ ^]^

The Wnt signaling pathway, encompassing Wnt ligands, receptors, intracellular components, transcription factors, and antagonists, plays a crucial role in bone development, formation, and homeostasis.^[^ ^6^ ^]^ Upon activation by Wnt ligands, the canonical Wnt signaling stabilizes β‐catenin, which translocates to the nucleus to activate target genes essential for osteoblast differentiation and function. Schnurri‐3 (SHN3), a large zinc finger adapter protein encoded by *Hivep3*, acts as a potent cell‐intrinsic negative regulator of osteoblast activity. SHN3 inhibits the Wnt signaling pathway by selectively suppressing the phosphorylation of specific ERK substrates such as p90RSK and GSK3β, rather than directly inactivating ERK itself. This regulation is critical because reducing ERK activity through the Mek1/2‐Het genotype reverses the high bone mass observed in SHN3 haploinsufficiency, indicating that SHN3 suppresses ERK activity in vivo.^[^ ^7^ ^]^ Consequently, the Wnt/SHN3 pathway has garnered significant attention due to its regulatory influence on osteoblast differentiation and bone mass maintenance.^[^ ^7^, ^8^ ^]^ Our prior studies discovered that mice deficient in SHN3 exhibit adult‐onset osteosclerosis, characterized by increased bone mass resulting from enhanced osteoblast activity.^[^ ^8^, ^9^ ^]^ More recently, SHN3 has been shown to play a crucial role in the treatment of bone‐fracture,^[^ ^9^, ^10^ ^]^ osteoporosis,^[^ ^8^, ^11^ ^]^ rheumatoid arthritis,^[^ ^12^ ^]^ and OI^[^ ^13^ ^]^ in animal models. Osteoblast‐lineage cells exhibit high sensitivity following reduced SHN3 expression levels. Notably, a mere 30% decrease in SHN3 mRNA levels in adult mice resulted in a significant, ≈50%, increase in bone mass.^[^ ^8^, ^14^ ^]^ Partial SHN3 inhibition can lead to significant increases in bone mass, facilitated through an increased rate of bone formation. This suggests that compounds capable of blocking SHN3 expression or activity serve as promising anabolic agents for the treatment of bone loss diseases. Bone‐targeting AAV or exosome‐mediated silencing of SHN3 expression^[^ ^8^, ^10^, ^11^ ^]^ marks the dawn of treating bone loss diseases. Still, currently, there are no literature reports on the targeted delivery of natural small molecules to silence SHN3. Targeting the Wnt/SHN3 pathway can serve as a powerful new strategy for treating bone loss diseases by enhancing bone formation.

**Scheme 1:**
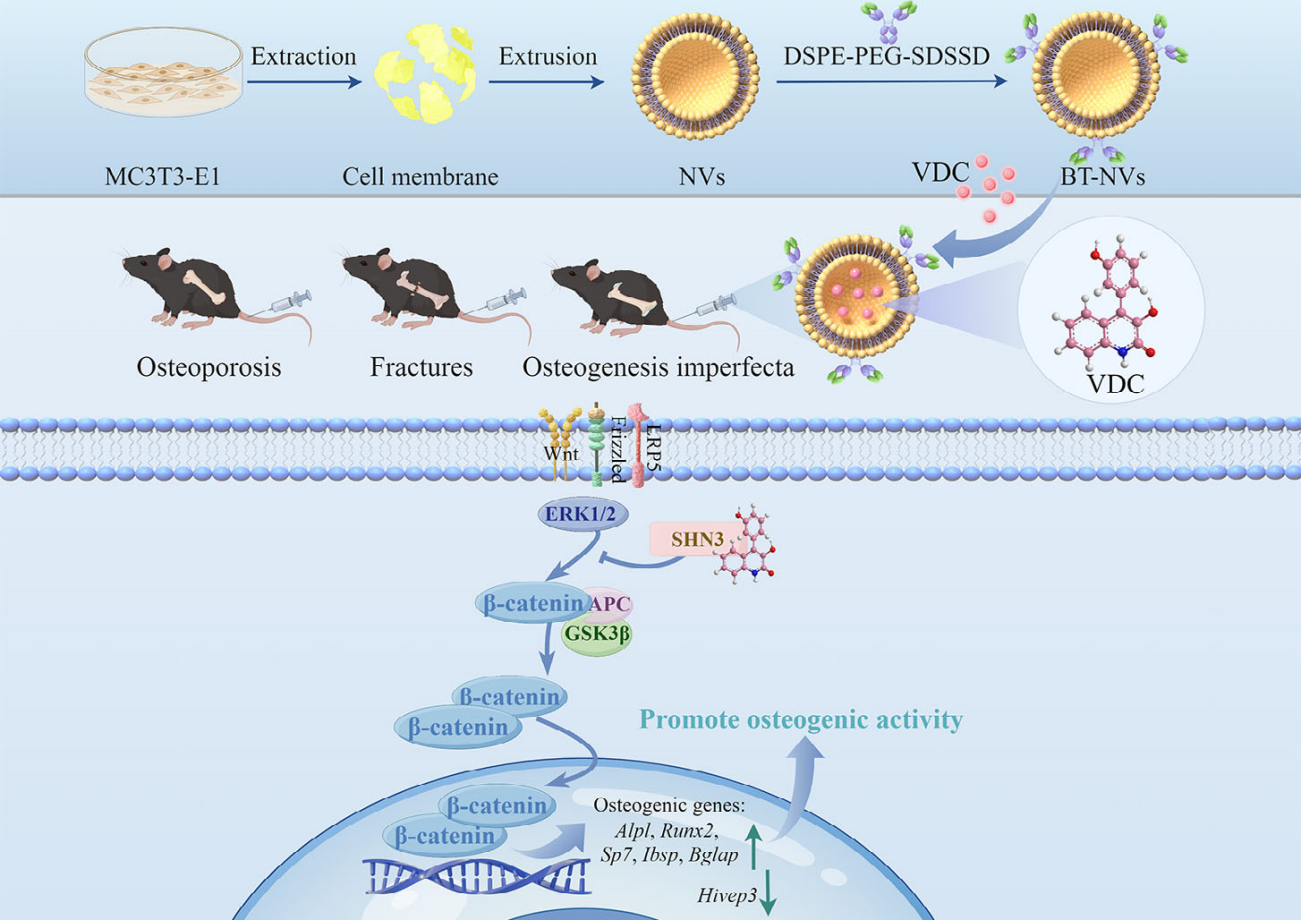
Schematic diagram of the potential mechanism by which viridicatol (VDC), derived from deep‐sea fungus, enhances osteoblastogenesis through modulation of the Wnt/SHN3 signaling pathway. Bone‐targeting nanovesicles (BT‐NVs) were engineered from MC3T3‐E1 cells using a multi‐step process, including ultrasonic lysis, density gradient centrifugation, and hydrophobic surface modification. VDC was subsequently loaded into BT‐NVs via ultrasonic encapsulation, improving targeted delivery to osteoblasts. Once delivered, VDC enhances osteogenic activity by regulating key mediator SHN3 in the Wnt signaling pathway, manifested by promoting the expression of key genes involved in osteogenic differentiation, such as upregulating the expression of *alkaline phosphatase (Alpl)*, *Runt‐related transcription factor 2* (*Runx2*), *Osterix* (*SP7*), *Integrin binding sialoprotein* (*Ibsp*), *Osteocalcin* (*Bglap*), and downregulating the expression of *human immunodeficiency virus type I enhancer binding protein 3 (Hivep3)*, ultimately promoting bone formation. This innovative approach holds significant promise for treating osteoporosis, facilitating fracture repair, and addressing osteogenesis imperfecta.

The deep‐sea environment with its structurally diverse array of secondary metabolites is promising reservoir of new drug candidates.^[^ ^15^ ^]^ Numerous marine drugs are currently applied in clinical trials including ziconotide, cytarabine, and trabectedin. Despite the significant potential of marine natural products as effective drugs for the treatment of bone loss diseases, research on these marine products is limited.^[^ ^16^ ^]^ In this study, the osteogenic differentiation ability of 251 compounds from deep‐sea‐derived fungi were investigated for osteogenic activity. Among these compounds, viridicatol (VDC), a viridicatin‐type alkaloids with a *m*‐hydroxyl groups, was found to promote early osteogenic differentiation and late mineralization, while inhibiting SHN3. Previous studies have revealed that VDC exhibits various biological activities, including anti‐inflammatory, antitumor, and antifungal activities. This compound has been biosynthesized for research given that it exhibits beneficial biological activity; moreover, the enzyme that catalyzes *meta* hydroxylation rarely occurs naturally.^[^ ^17^ ^]^ Despite these beneficial properties exhibited by VDC, its underlying mechanism in bone metabolic diseases, and its therapeutic potential remains unclear. Consequently, we first report that VDC directly targets SHN3 and exhibits strong osteogenic activity in vitro and in vivo by activating the Wnt/SHN3 signaling pathway. To validate VDC's therapeutic potential, we assessed the osteogenic efficacy of VDC in vivo models of osteoporosis, fracture, and OI. Given the severity of OI, we developed a novel bone‐targeting drug delivery system using modified homologous membrane derivatives, resulting in the creation of bone‐targeting nanovesicles (BT‐NVs) for drug delivery. This method allows for the precise delivery of VDC to bone tissue, thereby effectively suppressing the SHN3 gene and enhancing the treatment of OI (**Scheme** **1**).

## 2 Results

### 2.1 VDC Promotes Osteogenesis Differentiation and Inhibits *Hivep3* Expression

**Figure 1:**
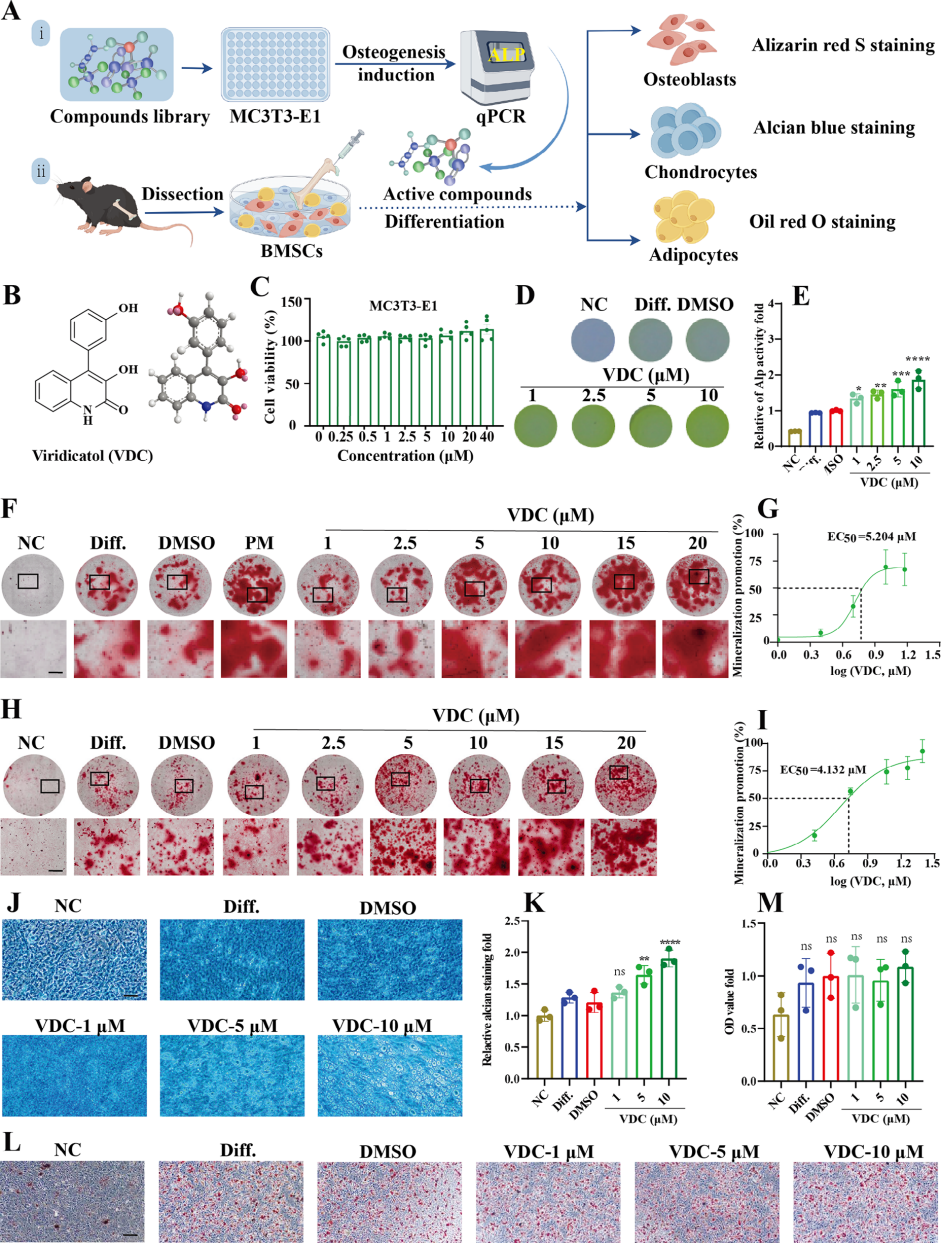
VDC promotes the osteogenesis activity of MC3T3‐E1 cells and BMSCs. A) A schematic figure showing the steps involved in screening for osteogenic activity of VDC. B) The 2D and 3D structures of VDC. C) The cytotoxicity of VDC (0–40 µm) among MC3T3‐E1 cells as determined by the CCK‐8 assay. (*n* = 5). D) Representative images of ALP activity analysis. Under osteogenesis induction conditions, the MC3T3‐E1 cells were treated with different concentrations of VDC (1, 2.5, 5, and 10 µm) for 5 days, and E) the quantitative analysis of VDC was calculated from the optical density values (*n* = 3). F) Representative images of alizarin red S staining. Under the osteogenesis induction conditions, the MC3T3‐E1 cells were treated with different concentrations of VDC (1, 2.5, 5, 10, 15, and 20 µm) or Purmorphamine (PM, 1 µm) for 21 days. The PM served as the positive control. Scale bar, 100 µm. G) The EC_50_ values of osteogenic mineralization (*n* = 5). H) Representative images of alizarin red S staining. The BMSCs were treated with different concentrations of VDC (1, 2.5, 5, 10, 15, and 20 µm) for 10 days under adipogenesis induction conditions. Scale bar, 100 µm. I) The EC_50_ values of osteogenic mineralization (*n* = 5). J) Representative images of alcian blue staining. The BMSCs were treated with different concentrations of VDC (1, 5, and 10 µm) for 21 days under adipogenesis induction conditions. Scale bar, 100 µm. K) The quantitative results of the alcian blue staining assay (*n* = 3). L) Representative images of Oil red O staining. The BMSCs were treated with different concentrations of VDC (1, 5, and 10 µm) for 10 days under adipogenesis induction conditions. Scale bar, 100 µm (*n* = 3). M) The quantitative results of Oil red O staining assay. Normal culture medium, differentiation medium, and 0.1% DMSO served as the negative control (NC), differentiation (Diff.), and solvent group (DMSO), respectively. Data represent mean ± SD, ns: not significant, *p < 0.05, **p < 0.01, ***p < 0.001, ****p < 0.0001 versus the DMSO group by one‐way ANOVA with Tukey's post‐hoc test.

To investigate the effects of natural marine products on osteogenic differentiation, we screened 251 compounds derived from deep‐sea fungi and measured alkaline phosphatase (Alp) activity as an indicator (**Figure** **1**A). 19 compounds showed notable osteogenic activity, with VDC being especially potent (Figure 1B). VDC significantly enhanced Alp activity while inhibiting *Hivep3* expression (Figure S1, Supporting Information). Additionally, VDC modulated the expression of key genes associated with various phases of osteogenesis, including *Runx2*, *Sp7*, *Atf4*, *Ibsp*, *Sema3a*, *Sema3e*, and *Spp1* (Figure S2, Supporting Information). Consequently, VDC was chosen for further investigation given its relative potency and yield. VDC exhibited robust Alp activity (Figure 1D,E) and osteoblast mineralization (Figure 1F) in a concentration‐dependent manner among the MC3T3‐E1 cells without cytotoxicity (Figure 1C). The EC_50_ value for promoting bone mineralization was 5.204 µm (Figure 1G). Bone‐derived mesenchymal stem cells (BMSCs) can provide an effective therapeutic strategy for preventing age‐related osteoporosis.^[^ ^18^ ^]^ Subsequently, we investigated the ability of VDC to directly induce BMSCs differentiation into osteoblasts, chondroblasts, and adipocytes. VDC significantly promoted the mineralization of BMSCs in a concentration‐dependent manner, with an EC_50_ value of 4.132 µm (Figure 1H,I). The adipogenic and chondrogenic differentiation of BMSCs exposed to the same concentrations of VDC were assessed through oil red O and alcian blue staining. The results demonstrated that VDC induced chondrogenic differentiation (Figure 1J,K), but had no effect on adipogenic differentiation among BMSCs (Figure 1L,M). These findings suggest that VDC effectively regulates BMSCs differentiation, promoting osteogenic mineralization and cartilage formation. Additionally, the TRAP staining results indicated that VDC did not exhibit concentration‐dependent inhibition of osteoclastogenesis activity within the concentration range of 1 to 10 µm. Consequently, we concluded that VDC exerts its effects primarily on osteoblasts rather than osteoclasts (Figure S3, Supporting Information).

### 2.2 VDC Induces Osteogenesis via Wnt Signaling

**Figure 2:**
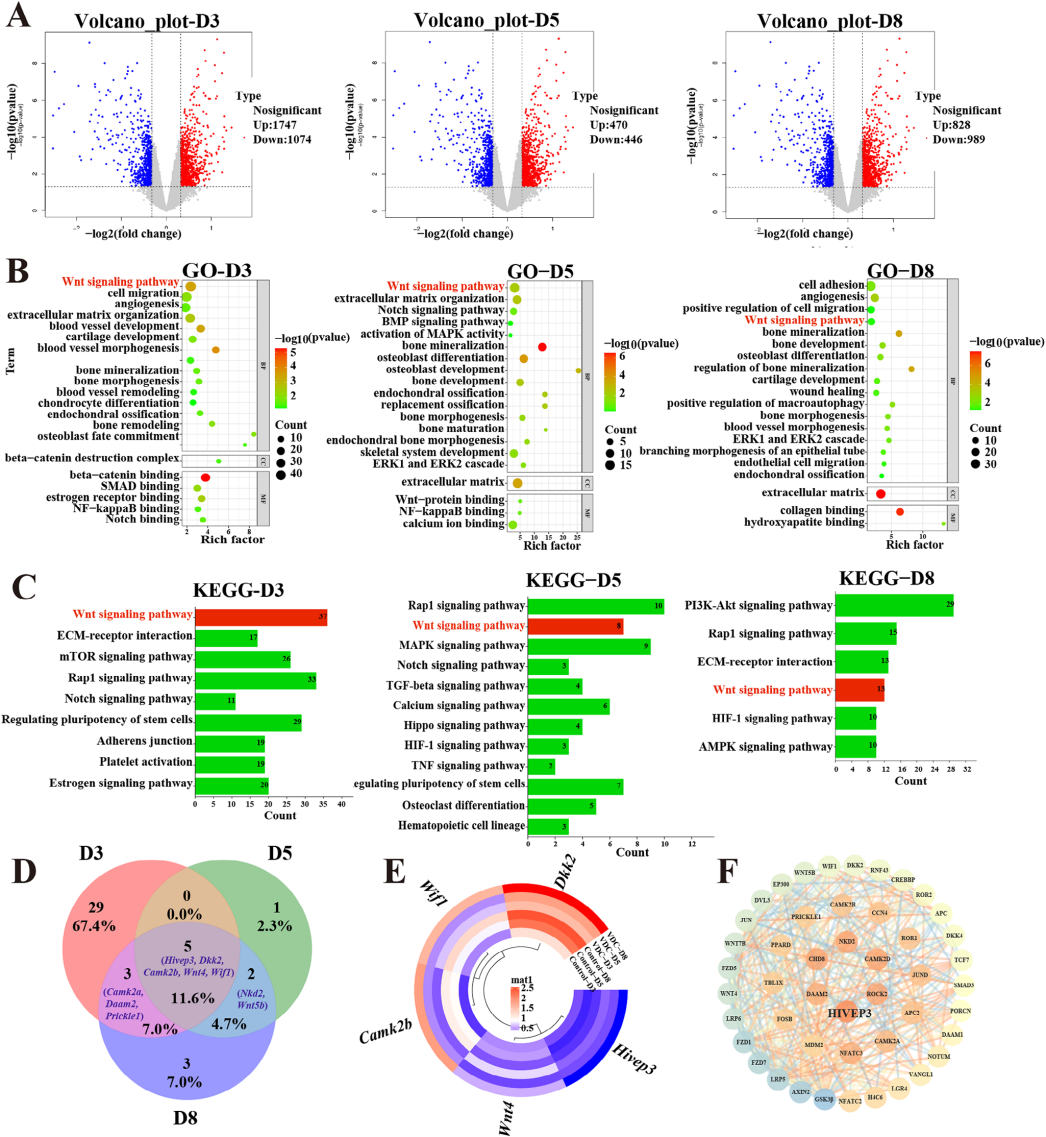
VDC induces osteogenesis via Wnt signaling pathway. A) Volcano plots showing the DEGs between control and VDC groups of 3, 5 and 8 days. Blue indicates downregulated genes; Red indicates upregulated genes. B) Gene Ontology (GO) enrichment analyses of the DEGs in RNA‐seq data showing the key upregulated biological process in VDC groups of 3, 5, and 8 days. C) The KEGG analysis results. D) Venn diagram showing the overlapping DEGs of the Wnt signaling pathway at 3, 5, and 8 days. E) Heatmap illustrating the overlap DEGs of the Wnt signaling pathway at 3, 5, and 8 days. F) The PPI regulatory network of the Wnt signaling pathway.

RNA‐seq was employed to explore the mechanisms underlying osteoblastogenesis in MC3T3‐E1 cells treated with or without VDC for 3, 5, and 8 days. The volcano plot analysis revealed significant differential expression of genes (DEGs) in VDC‐treated cells compared to the control cells at various time points. Specifically, 1747, 470, and 828 genes were upregulated on days 3, 5, and 8, respectively, whereas 1074, 446, and 989 genes were downregulated on the same days (**Figure** **2**A). To illustrate the function of DEGs, gene ontology (GO) analysis was performed on three levels: molecular function, cellular component, and biological process. The VDC‐treated group showed significant enrichment in pathways related to the Wnt signaling pathway, bone mineralization, ossification, and bone morphogenesis, in comparison to the control group on days 3, 5, and 8. Notably, the functions of the Wnt signaling pathway throughout the process, along with its optimal *P* and richness, are vital (Figure 2B). Kyoto Encyclopedia of Genes and Genomes (KEGG) analysis was conducted to explore the regulated pathways involving DEGs. The mechanisms of anti‐osteoporosis were predominant in the Wnt, PIK3‐AKT, Notch, mTOR, and MAPK signaling pathways (Figure 2C). Based on the results from GO, KEGG, and disease‐related biological knowledge analyses, we postulated that the Wnt signaling pathway may be the primary mediator of the VDC treatment effects on skeletal diseases. The Venn diagram (Figure 2D) was used to identify the intersection of DEGs within the Wnt signaling pathway at days 3, 5, and 8. The analysis revealed that *Hivep3*, *Dkk2*, *Camk2b*, *Wnt4*, and *Wif1* were consistently differentially expressed across these time points. During osteogenic differentiation, *Hivep3*, *Dkk2*, *Camk2b*, *Wnt4*, and *Wif1* show dynamic expression changes crucial for osteoblast regulation.^[^ ^7^, ^19^ ^]^ *Hivep3* influences ERK activity in Wnt signaling, affecting osteoblast differentiation. *Dkk2* modulates bone homeostasis by inhibiting Wnt signaling via LRP5/6. *Camk2b* interacts with Wnt pathways through calcium signaling to regulate bone metabolism. *Wnt4* promotes osteoblastogenesis by activating Wnt signaling, while *Wif1* inhibits it by binding to Wnt ligands. Together, these genes regulate bone metabolism and contribute to bone mineralization, ossification, and morphogenesis through VDC‐mediated signaling, as shown by GO analysis. The heatmap illustrating the DEGs at the intersection of the three‐time points is presented in Figure 2E. VDC treatment led to upregulation of *Dkk2*, *Camk2b*, *Wnt4*, and *Wif1*, while inhibiting the expression of *Hivep3*. GO and KEGG analysis revealed significant activation of the Wnt signaling pathway on days 3, 5, and 8, suggesting it may mediate VDC treatment effects on skeletal diseases. After deduplicating DEGs (37 on day 3, 8 on day 5, and 13 on day 8), a protein‐protein interaction (PPI) network diagram was constructed to investigate the crucial protein targets within the Wnt signaling pathway at these specified time points. The findings from the analyses of the PPI regulatory networks (Figure 2F) revealed interactions among potential therapeutic targets at these time points, comprising 47 nodes and 280 edges. Notably, *Hivep3* exhibited the strongest correlation and occupied the central position within the network graph. Consequently, *Hivep3* was identified as the primary target of VDC within the Wnt signaling pathway.

### 2.3 VDC Stimulates the Expression of Osteoblast‐Specific Proteins by Activating the Wnt/SHN3 Signaling Pathway

**Figure 3:**
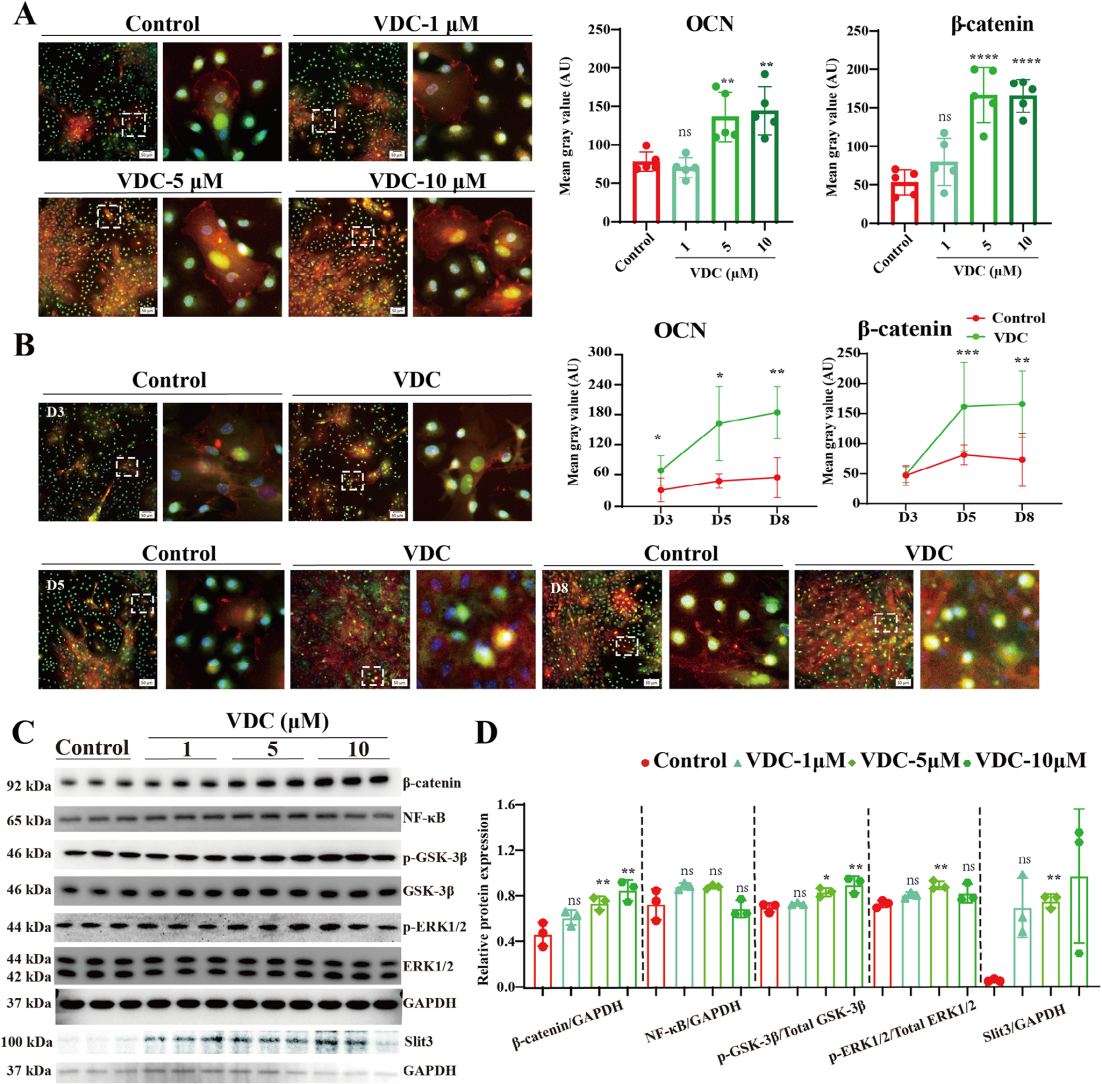
VDC stimulates the expression of osteoblast‐specific proteins by the Wnt/SHN3 pathway. A) Representative images and corresponding quantitative analysis of immunofluorescence staining results of OCN and β‐catenin treated with 1, 5, and 10 µm VDC for 5 days (*n* = 5). Scale bar, 50 µm. B) Representative images and quantitative analysis of immunofluorescence staining of OCN and β‐catenin treated with VDC (5 µm) for 3 days, 5 days, and 8 days (*n* = 5). Scale bar, 50 µm. C) Representative images of western blot showing the expression levels of β‐catenin, NF‐κB, GSK‐3β, phosphorylated (p)‐GSK‐3β, ERK1/2, p‐ERK1/2, Slit3 and normalized to the expression of GAPDH. Under induction conditions (50 µg mL^−1^ ascorbic acid and 5 mm β‐glycerophosphate), MC3T3‐E1 cells were cultured with VDC (1, 5, 10 µm) or 0.1% DMSO (control group) for 5 days. The solvent control group (Control) was treated with 0.1% DMSO (*n* = 3). D) Quantitative analysis of the relative protein expression for β‐catenin, NF‐κB, p‐GSK‐3β and p‐ERK1/2 and Slit3. Data represent mean ± SD, ns: not significant, *p < 0.05, **p < 0.01, ***p < 0.001, ****p < 0.0001 versus the Control group by one‐way ANOVA with Tukey's post‐hoc test.

Our previous studies demonstrated that SHN3 negatively regulates bone mass by inhibiting ERK activity while also activating downstream β‐catenin via GSK‐3β to promote osteogenesis.^[^ ^20^ ^]^ To validate that the Wnt/SHN3 signaling pathway was activated through VDC treatment in BMSCs, we performed immunofluorescence staining to detect the level of β‐catenin and its co‐localization with osteocalcin (OCN) in BMSCs. Immunofluorescence results showed that the OCN and β‐catenin levels were significantly upregulated in BMSCs treated with VDC, and it did not dependent on concentration or time (**Figure** **3**A,B).

Additionally, SHN3 controls GSK3β activity and the levels of β‐catenin and via ERK in Wnt signaling^[^ ^5^, ^7^ ^]^ And the NF‐κB controls the differentiation or activity of osteoblasts.^[^ ^21^ ^]^ Therefore, we used western blot analysis to assess the effects of VDC on the expression levels of β‐catenin, NF‐κB, GSK‐3β, phosphorylated(p)‐GSK‐3β, ERK1/2, p‐ERK1/2, Slit3, and SHN3. Treatment with VDC for 5 days in BMSCs resulted in the upregulation of β‐catenin, p‐GSK‐3β, and p‐ERK1/2 expression levels, while the expression levels of NF‐κB, total ERK1/2, and GSK‐3β remained unchanged (Figure 3C,D). And it also revealed that VDC significantly inhibits SHN3 protein expression, thereby validating its mechanistic role in regulating SHN3 activity (Figure S4, Supporting Information). The significant modulation of these proteins by VDC underscores its role in regulating osteogenic activity via the Wnt/SHN3 signaling pathway.

### 2.4 VDC‐Mediated Silencing of SHN3 Promotes Osteogenic Activity

**Figure 4:**
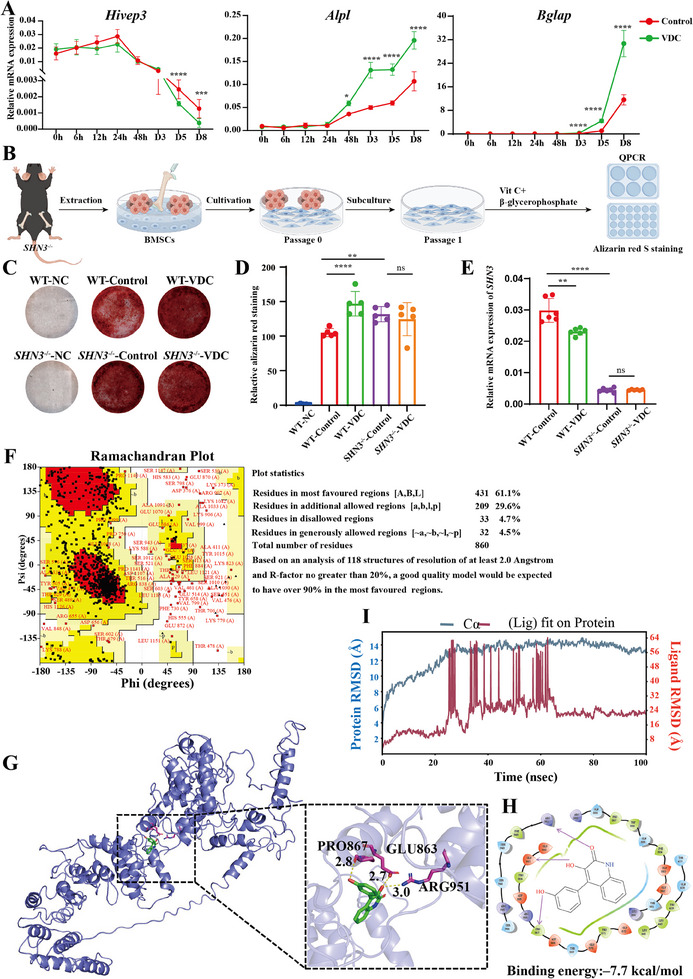
SHN3 is a key target for VDC‐induced osteoblast activity. A) The effect of VDC on the expression levels of key genes *Hivep3*, *Alpl*, and *Bglap* among MC3T3‐E1 cells. The VDC (5 µm) was added to MC3T3‐E1 cells and cultured for the indicated durations (0 h, 6 h, 12 h, 24 h, 48 h, 3 days, 5 days, 8 days) under induction conditions (*n* = 6). B) A diagram showing the BMSCs extraction procedure. (C) Representative images of alizarin red S staining assay for osteogenic mineralization in BMSCs. The VDC (5 µm) was added to BMSCs for induction for 8 days under osteogenic induction conditions, which were subjected to alizarin red staining and microscopic images were recorded. D) Quantitative analysis of Alizarin red staining (*n* = 5). E) qPCR validation of the knockout efficiency of *SHN3* and the inhibitory effect of VDC on *SHN3*. The VDC (5 µm) was added in BMSCs for induction 5 days under osteogenic induction conditions (*n* = 6). F) The laplace diagram was produced directly by the SAVES server. G) The binding modes of VDC and SHN3 in a 3D workspace cartoon, as well as their detailed enlarged images. H) 2D combination mode diagram of VDC with SHN3. The H‐bonding was expressed as a pink arrow. Normal culture medium and 0.1% DMSO served as the negative control group (NC) and solvent control group (control), respectively. I) Molecular dynamics simulation analysis of SHN3 and VDC. Root mean square deviation (RMSD) plot of VDC with respect to SHN3 protein. Data represent mean ± SD, ns: not significant, *p < 0.05, **p < 0.01, ***p < 0.001, and ****p < 0.0001 versus the DMSO group by one‐way ANOVA with Tukey's post‐hoc test.

To further validate the regulatory effect of VDC on *Hivep3*, we used quantitative polymerase chain reaction (qPCR) to assess the expression level of *Hivep3* at time points of 0, 6, 12, 24, and 48 h, as well as at 3, 5, and 8 days. Under osteogenic induction conditions, the expression profiles of the *Hivep3* gene initially increased gradually, before sharply decreasing in the early stages of osteogenic differentiation. This observation demonstrated the negative regulatory role of *Hivep3* on the osteogenic differentiation process of osteoblasts. Upon exposure to VDC (5 µm) for 3, 5, and 8 days, VDC exhibited a time‐dependent inhibition of *Hivep3* expression, while promoting the expression of *alkaline phosphatase* (*Alpl*, marker gene for early osteogenic differentiation) and *osteocalcin* (*Bglap*, marker gene for late osteogenic differentiation) in a time‐dependent manner (**Figure** **4**A).

To further explore the role of SHN3 in VDC‐induced osteoblast differentiation, BMSCs were extracted from 3‐week‐old SHN3^−/−^ knockout and wild‐type (WT) mice and cultured in vitro. The cells were treated with 5 µm VDC. qPCR and alizarin red S staining analyses were performed (Figure 4B). In the absence of SHN3, both KO control and WT‐VDC significantly promoted the osteogenic mineralization of BMSCs compared to WT control. However, VDC's osteogenic mineralization effect on BMSCs was diminished without SHN3, suggesting that SHN3 is crucial for VDC‐induced osteoblast mineralization (Figure 4C–E).

In a study by Jones,^[^ ^7^ ^]^ a D‐domain motif within SHN3 was found to mediate the interaction with and inhibition of ERK activity and osteoblast differentiation. However, the 3D structure of SHN3 remains currently unknown due to its high molecular weight (>260 kDa) and low expression level. To explore whether VDC can bind with SHN3, we simulated SHN3 protein structure using the de novo prediction method. We focused on amino acids 327–1183 (NCBI Reference Sequence: NP_0 011 21186.1) of SHN3 for prediction and evaluated the model quality by constructing Laplace plots. The Laplace diagram, generated through the SAVES server, indicated that 90.7% of the protein residues fell within the allowed red and yellow maximum regions, demonstrating high reliability for molecular docking (Figure 4F). Our analysis focused on the protein‐ligand interactions, identifying and categorizing all functional residues based on their specific interactions. Various groups of residues were found to participate in the interactions between SHN3 and VDC, including the hydrogen bond formed by GLU863 of SHN3 with the ligand. These interactive forces generated a binding energy of −7.7 kcal mol^−1^ for the protein‐ligand complex, indicating it was a strong binding force (Figure 4G,H). Moreover, molecular dynamics simulations to investigate the interactions between VDC and SHN3.The root mean square deviation (RMSD) plot illustrates the SHN3 protein remained relatively stable, whereas the ligand exhibited fluctuations before stabilizing in the final RMSD. Post‐dynamic simulation, a more stable binding conformation was established on the original basis, indicating enhanced conformational and interaction stability of the SHN3 and VDC complexes (Figure 4I). In comparison to the initial binding conformation, VDC has experienced a certain degree of deviation (Figure S5A, Supporting Information). This shift suggests dynamic adjustments in the ligand's position relative to the protein during the simulation. The Ligand Root Mean Square Fluctuation (L‐RMSF) is a valuable metric for characterizing positional changes of ligand atoms, while the Root Mean Square Fluctuation (RMSF) provides insights into local flexibility along the protein backbone. As shown in Figure S5B (Supporting Information), the RMSF values of amino acid residues involved in interactions with the small molecule (highlighted with green lines) are generally higher, indicating significant conformational rearrangements during binding. Consistent with this observation, the L‐RMSF values of the small molecule's atoms are also relatively high (Figure S5C, Supporting Information), further supporting the notion of substantial mobility and structural adaptation during the interaction. Throughout the simulation, protein‐ligand interactions within 5 Å of VDC were monitored, as illustrated in the Interaction Fractions plot (Figure S5D, Supporting Information). These interactions include hydrogen bonds, hydrophobic contacts, and water‐mediated bridges, underscoring the complexity and diversity of the binding mechanism. Notably, several surrounding amino acids—GLN 438, LEU 440, GLU 863, PRO 873, LYS 880, GLU 883, GLU 935, HIS 984, and GLU 987—played critical roles in stabilizing the binding of VDC. This stability supports the hypothesis that VDC effectively targets SHN3, thereby modulating the Wnt/SHN3 pathway and promoting osteogenic differentiation.

### 2.5 VDC Ameliorates OVX‐Induced Bone Loss and Promotes Fracture Healing

The absence of SHN3 effectively restores bone remodeling processes, mitigating bone loss associated with osteoporosis, facilitating fracture repair, and treating OI.^[^ ^8^, ^9^, ^10^, ^11^, ^12^, ^22^ ^]^ To further investigate whether VDC‐mediated silencing of SHN3 offers therapeutic benefits for these osteolytic diseases, we assessed the osteogenic effects of VDC in vivo using ovariectomized (OVX), fractured, and OI mouse models.

**Figure 5:**
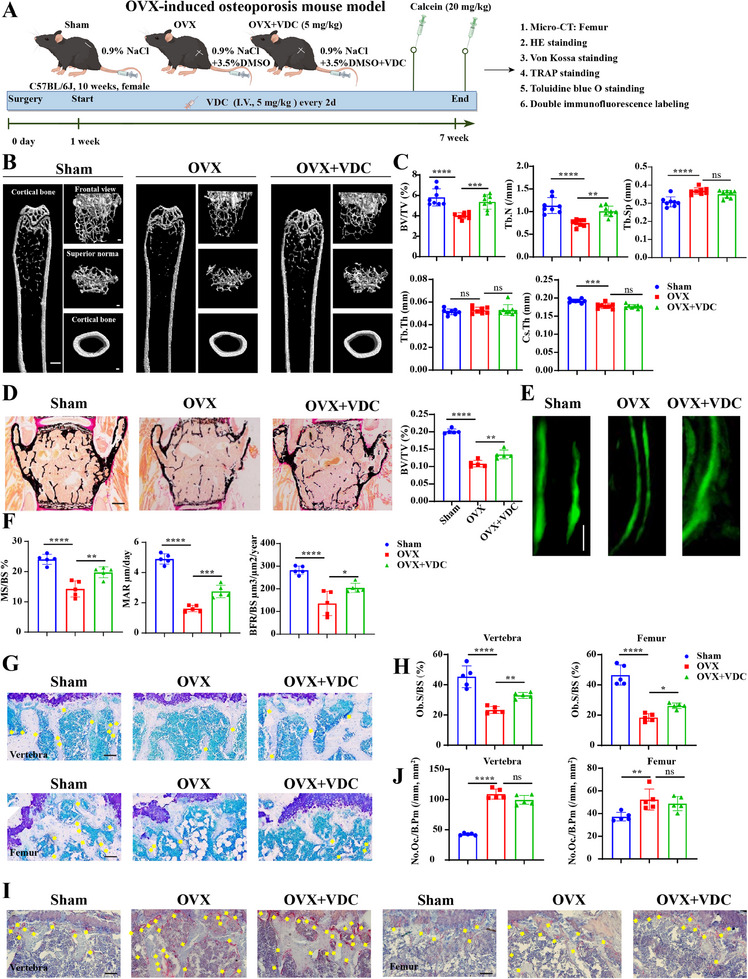
VDC delays bone loss in a mouse model of osteoporosis. A) Schematic diagram of the mouse OVX model. B) 3D reconstructed images of mouse femur after Micro‐CT scanning. Scale bar, 1 mm (long femur) and 100 µm (frontal view, superior norma and cortical bone). C) Quantitative measurements of bone microstructure‐related parameters: BV/TV, Tb.N, Tb.Sp, Tb.Th, and Cs.Th (*n* = 8). D) Representative images and BV/TV quantitative of Von Kossa stained bone tissue in the fifth lumbar spine. Scale bar, 250 µm. E) Representative fluorescence images of the double‐labeled resin sections of vertebral bone with calcein. Scale bar, 25 µm (*n* = 5). F) Quantitative analysis of the dynamic bone reconstruction: MS/BS, MAR, and BFR/BS. G) Representative images of the decalcified bone stained with Toluidine blue. Scale bar, 100 µm. H) Quantitative analysis of Toluidine blue staining results for the lumbar spine and femur (*n* = 5). I) Representative images of the decalcified bone stained with TRAP staining. Scale bar, 100 µm. J) Quantitative measurements of TRAP staining of the lumbar spine and femur (*n* = 5). Data represent mean ± SD, ns: not significant, *p < 0.05, **p < 0.01, ***p < 0.001, and ****p < 0.0001 versus the OVX group by one‐way ANOVA with Tukey's post‐hoc test.

VDC was tested for its effects on estrogen‐induced osteoporosis in mice after ovariectomy. The schematic diagram of the OVX model is shown in (**Figure** **5**A). C57BL/6J mice were injected with VDC (5 mg kg^−1^) or DMSO for 6 weeks, with no significant weight changes or deaths observed (Figure S6A, Supporting Information). HE staining revealed no abnormalities in the liver, spleen, and kidneys, indicating VDC was safe for use (Figure S6B, Supporting Information). The OVX surgery resulted in uterine atrophy and weight loss, successfully modeling osteoporosis (Figure S6C,D, Supporting Information).

CT scan data of femurs indicated that the OVX+VDC group exhibited higher bone volume fraction (BV/TV) and trabecular bone number (Tb. N) compared to the OVX‐only group (Figure 5B,C). Moreover, Von Kossa staining of the 5th lumbar spine bone tissue showed increased bone mass with VDC treatment (Figure 5D). Fluorescence double labeling results demonstrated improvements in the mineralized surface to bone surface ratio (MS/BS), mineral appositional rate (MAR), and bone formation rate to bone surface ratio (BFR/BS) in the OVX+VDC group (Figure 5E,F). These dynamic bone parameters demonstrate an overall increase in bone formation after VDC treatment. Additionally, Toluidine blue (Figure 5G) and TRAP staining (Figure 5I) revealed that VDC enhanced the osteoblast surface/bone surface ratio (Ob.S/BS) in the 5th lumbar vertebrae and femur (Figure 5H), with no significant effect on the osteoclast count/bone surface ratio (N.Oc.S/BS) (Figure 5J). Collectively, these static and dynamic bone parameters suggest that VDC stimulated bone formation in vivo by enhancing osteoblast activity, thereby preventing bone loss in OVX mice.

**Figure 6:**
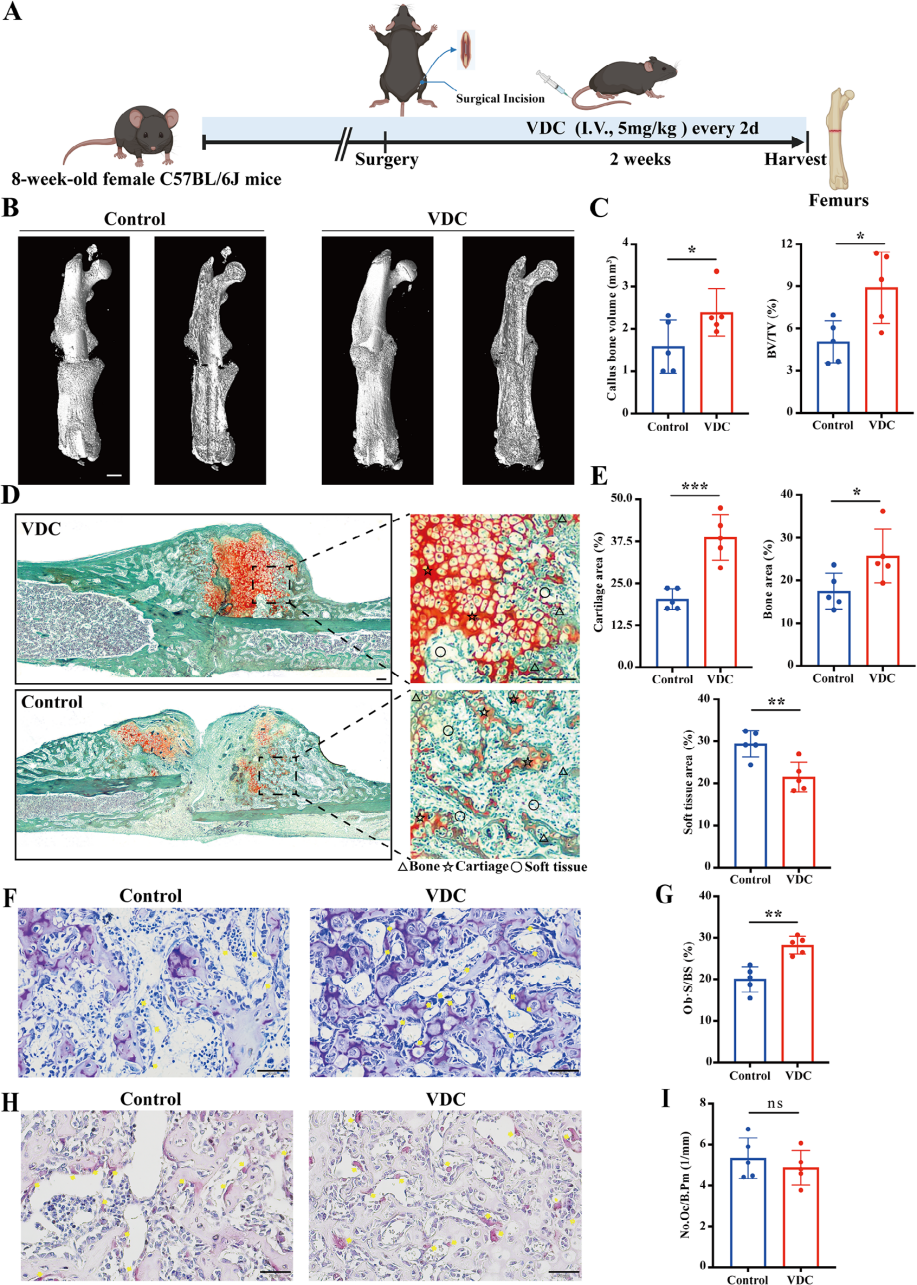
VDC promotes fracture healing. A) A schematic diagram of the mouse fracture model. B) Representative 3D reconstructed images obtained through Micro‐CT scanning of mouse femoral fractures. Scale bar, 1 mm. C) Quantification of callus volume and BV/TV of fractures (*n* = 5). D) Representative images showing the results of the Safranin O/Fast Green staining. Scale bar, 1 mm (Overall view) and 200 µm (enlarged view). E) Quantitative analysis of bone area, soft tissue area, and chondrocyte area (*n* = 5). F) Representative images of the decalcified bone stained with Toluidine blue. Scale bar, 50 µm (*n* = 5). G) Quantitative measurements of Toluidine blue staining (*n* = 5). H) Representative images of decalcified bone subjected to TRAP staining. Scale bar, 50 µm. I) Quantitative measurements of TRAP staining (*n* = 5). Data represent mean ± SD, ns: not significant, *p < 0.05, **p < 0.01, ***p < 0.001, and ****p < 0.0001 versus the control group (0.1%DMSO+PBS) by an unpaired two‐tailed Student's t‐test.

To evaluate the pharmacological effects of VDC on fractures, we established an open fracture model in 8‐week‐old female C57BL/6J mice. The mice then underwent postoperative administration of VDC‐containing matrix gel (5 mg kg^−1^) or 3.5% DMSO matrix gel with injections every 2 days for 14 days (**Figure** **6**A). CT scans showed that VDC significantly increased callus volume and bone mass (Figure 6B,C). The results of Safranin O/Fast Green staining further demonstrated that VDC increased cartilage and bone areas, underscoring its potential to promote fracture healing (Figure 6D,E). Additionally, results from toluidine blue and TRAP staining of fractures demonstrated that VDC primarily exerts its effects on osteoblasts rather than osteoclasts (Figure 6F–I).

### 2.6 Targeted Delivery of VDC for Treating OI

To investigate the therapeutic potential of VDC in treating OI, a *Col1a2* ^oim/oim^ mice model was used. This model has been demonstrated to be appropriate for cases of moderate to severe OI.^[^ ^13^, ^23^ ^]^ Due to the severity of OI mice (OIM), achieving the desired therapeutic effect can be challenging. To enhance tissue accumulation and the efficacy of VDC, we explored the development of a bone‐targeting system.

Cellular nanovesicles (NVs), fabricated through direct secretion or physicochemical methods, are increasingly favored for drug delivery due to their excellent biocompatibility.^[^ ^24^ ^]^ Exosomes, a naturally secreted NV, are extensively investigated due to their structure, composition, and unique role in intercellular communication. However, despite their promising therapeutic potential, the clinical application of exosomes remains constrained by complex production processes and low yields. However, advancements in nanotechnology have enabled the production of NVs through various physical and chemical methods, resulting in significantly higher yields compared to naturally secreted exosomes.^[^ ^25^ ^]^ Moreover, effective targeted modifications of these NVs enhance delivery efficiency to specific organs and maximize their carrier potential.^[^ ^26^ ^]^ In this study, we used SDSSD (Ser, Asp, Ser, Ser, Asp), a proven bone‐targeting peptide, to engineer BT‐NVs for the precise delivery of VDC.^[^ ^27^ ^]^

**Figure 7:**
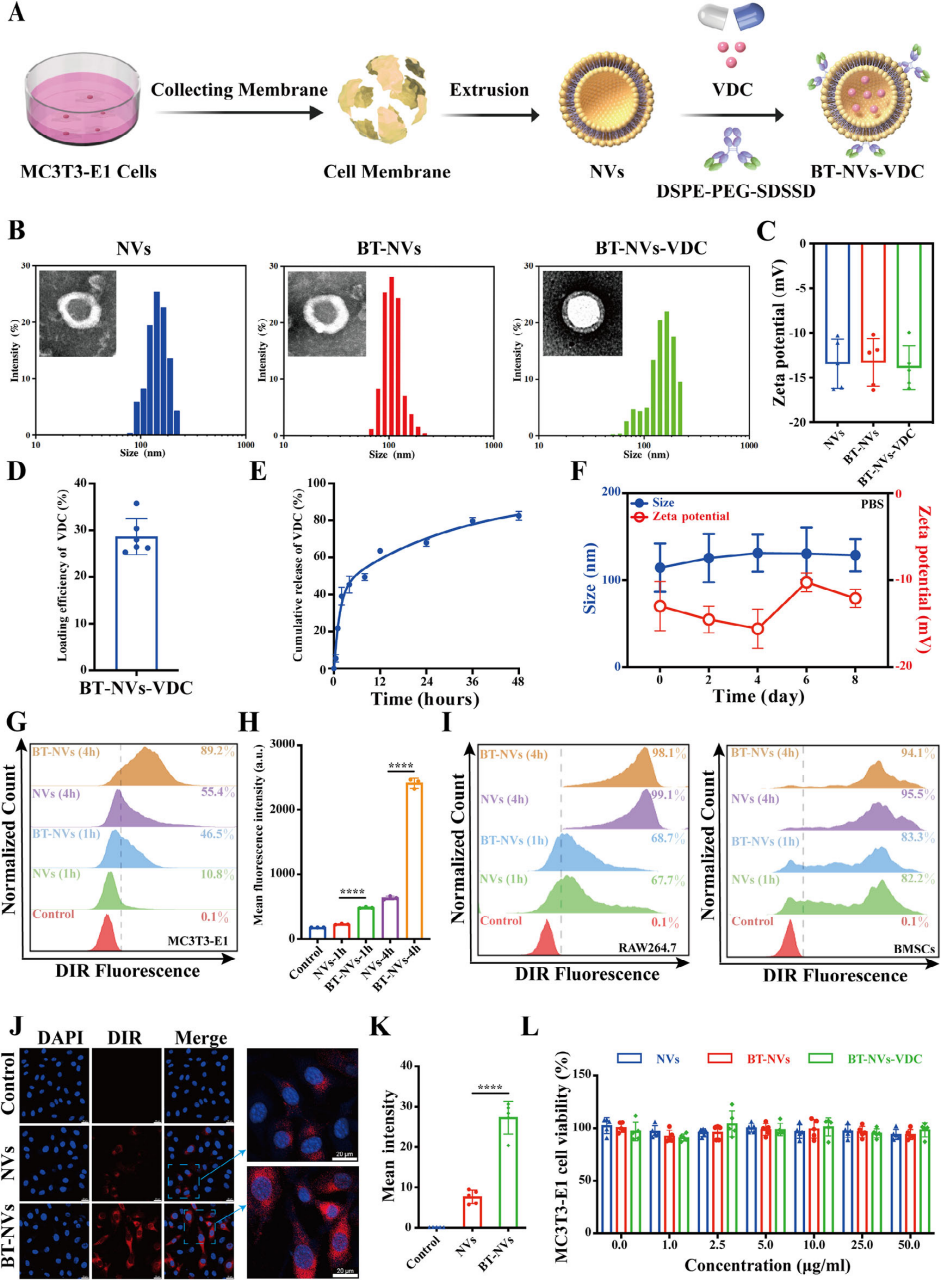
Preparation and characterization of BT‐NVs‐VDC. A) A schematic representation of the preparation process of BT‐NVs‐VDC. B) Size distribution and TEM images of NVs, BT‐NVs and BT‐NVs‐VDC. C) The average surface zeta potential of NVs, BT‐NVs and BT‐NVs‐VDC (*n* = 5). D) Loading efficiency of VDC in BT‐NVs (*n* = 6). E) In vitro drug release profiles under PBS (pH 7.4) conditions (*n* = 3). F) The stability of BT‐NVs. Changes in size and zeta potential of BT‐NVs in PBS during the 8 days of treatment (*n* = 3). G–I) Flow cytometry analysis of DiR‐labeled NVs or BT‐NVs binding to MC3T3‐E1, RAW264.7 and BMSC cells after a 1 or 4 h incubation (*n* = 3). J,K) Representative confocal microscopy images of MC3T3‐E1 cells incubated with DiR‐labeled NVs or BT‐NVs for 4 h and the mean fluorescence intensity analysis. Scale bar, 20 µm. (*n* = 5). L) Cytotoxicity of NVs, BT‐NVs, and BT‐NVs‐VDC against MC3T3‐E1 cells after a 24 h incubation at indicated concentrations (*n* = 5). Data represent mean ± SD, ns: not significant, *p < 0.05, **p < 0.01, ***p < 0.001, and ****p < 0.0001 by one‐way ANOVA with Tukey's post‐hoc test.

BT‐NVs extracted from MC3T3‐E1 cells were produced through ultrasonic lysis, followed by density gradient centrifugation, and then hydrophobic modification. Subsequently, VDC was loaded into BT‐NV using ultrasonic to prepare BT‐NVs‐VDC (**Figure** **7**A). The characterization of BT‐NVs‐VDC was then performed. Transmission electron microscopy (TEM) revealed that NVs, BT‐NVs, and BT‐NVs‐VDC exhibited cup‐shaped morphology. Notably, the BT‐NVs‐VDC group exhibited increased fluorescence compared to the other groups, indicating successful VDC loading. Dynamic light scattering (DLS) analysis showed that the NVs were NVs with an average size of 127.60 nm and a zeta potential of ≈−13.87 mV. Additionally, no significant changes were observed in size or zeta potential following subsequent modifications (Figure 7B,C). The VDC‐loading efficiency was ≈28.67% and ≈82.51% of VDC was released from PBS after 48 h (Figure 7D,E). The stability of BT‐NVs in vitro and in vivo was assessed using PBS supplemented with 10% FBS. The results indicated that BT‐NVs remained stable over 8 days (Figure 7F; Figure S7, Supporting Information).

We then investigated the efficiency of the bone‐targeting peptide by comparing the uptake of DIR‐labeled NVs and BT‐NVs in MC3T3‐E1 cells after incubation for 1 or 4 h. Flow cytometry revealed stronger fluorescent signals in the BT‐NVs group compared to the NVs group (Figure 7G,H). However, no significant differences were observed in BMSCs and RAW264.7 cells (Figure 7I). Subsequently, osteoblast‐specific uptake was further validated using confocal laser scanning microscopy (CLSM) (Figure 7J,K). CCK‐8 assays indicated that these three NVs exhibited optimal cytocompatibility (Figure 7L). In summary, we have developed an effective bone‐targeting delivery system.

**Figure 8:**
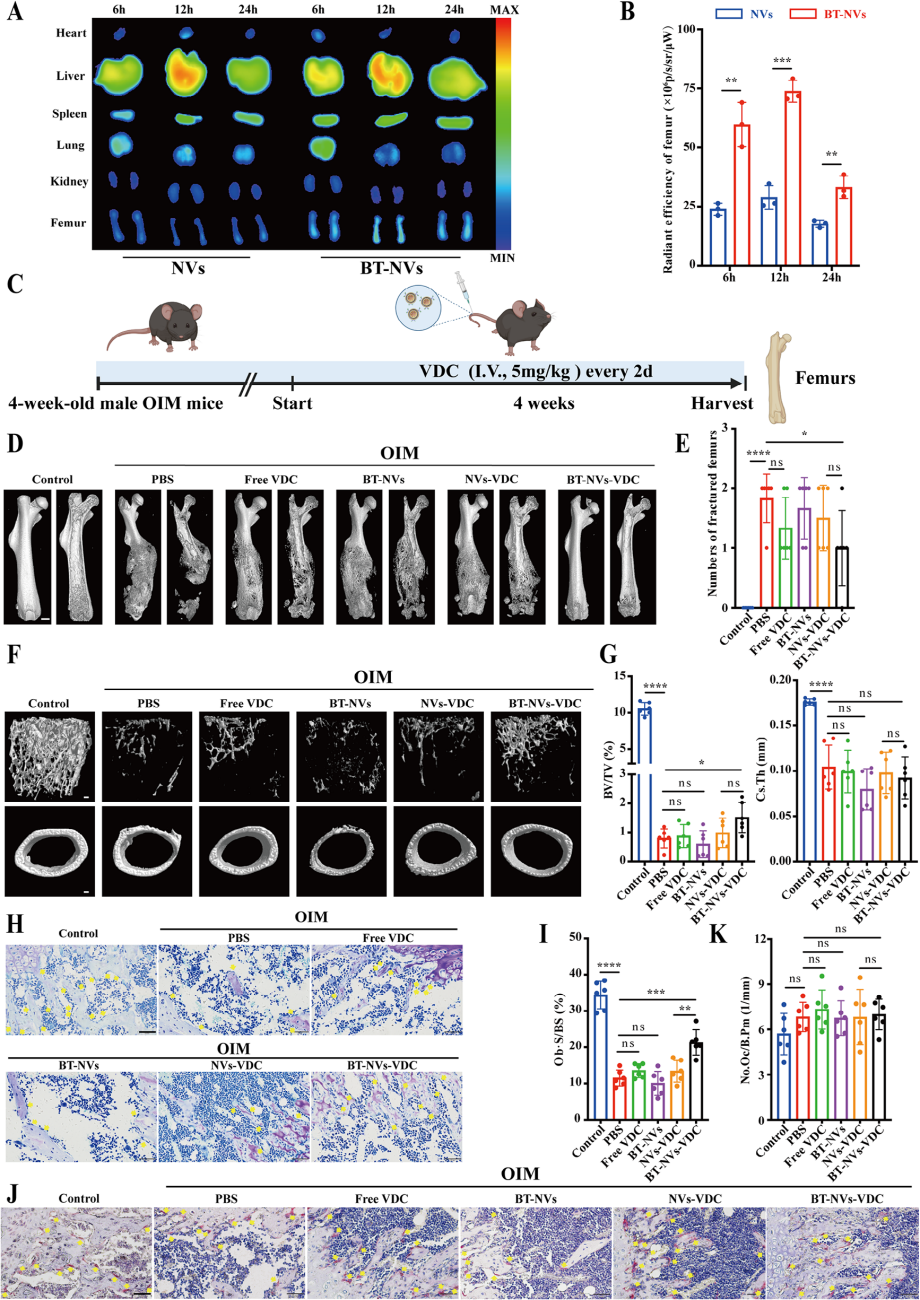
VDC delays bone loss in a mouse model of osteogenesis imperfecta and reduces the occurrence of spontaneous fractures. A) The biodistribution of DiR‐labelled NVs or BT‐NVs at a specified time points after intravenous injection. B) Radiant efficiency of DiR‐labelled NVs or BT‐NVs in femurs (*n* = 3). C) Schematic diagram of the mouse OI model. D) Reconstruction of µCT data reflected spontaneous bone fracture in OIM groups. Scale bar, 1 mm. E) The number of spontaneous bone fracture femurs in the OIM groups. F) Four weeks after intravenous injection into 1‐month‐old male mice, femoral trabecular bone mass was evaluated with µCT. Scale bar, 100 µm. G) Representative 3D reconstructions and corresponding quantitative analysis are presented (*n* = 5). H) Representative images of decalcified bone subjected to the Toluidine blue staining. Scale bar, 50 µm. I) Quantitative measurements of Toluidine blue staining in the femurs, (*n* = 5). J) Representative images of the decalcified bone stained with TRAP staining. Scale bar, 50 µm. K) Quantitative measurements of TRAP staining in the femurs (*n* = 5). Data represent mean ± SD, ns: not significant, *p < 0.05, **p < 0.01, ***p < 0.001, and ****p < 0.0001 by one‐way ANOVA with Tukey's post‐hoc test.

Before OIM treatment, we assessed the in vivo distribution effects of NVs and BT‐NVs. Analysis of the IVIS Spectrum revealed that the bone‐targeted modification was effective, increased accumulation in bone tissues such as the femurs (**Figure** **8**A,B; Figure S8, Supporting Information). Based on these observations, we proceeded with in vivo experiments (Figure 8C). As anticipated, the OIM exhibited a severe osteopenic phenotype and spontaneous bone fractures compared to the WT group during the 4‐week treatment period. Additionally, the therapeutic effects of free VDC and NVs‐VDC were limited, due to insufficient effective concentrations at the target organs caused by blood diffusion during long‐term circulation. Conversely, BT‐NVs‐VDC significantly mitigated the osteopenic symptoms and reduced spontaneous fracture occurrences in OIM (Figure 8D‐G; Figure S9, Supporting Information). Subsequent studies further validated the effect of VDC on osteoblasts in OIM (Figure 8H–J). Collectively, these bone parameters indicate that VDC promotes bone formation by enhancing osteoblast activity in vivo, thereby mitigating bone loss and reducing the incidence of spontaneous fractures in OIM.

## 3 Discussion

The skeletal system of healthy adults is regulated by a dynamic equilibrium maintained by osteoclasts and osteoblasts. Osteoblasts play a crucial role in bone reconstruction, and their quantity as well as functionality is closely linked to various osteolytic diseases, including osteoporosis, osteonecrosis, rheumatoid arthritis, and osteoarthritis. Based on current literature reviews^[^ ^15^, ^16^, ^28^ ^]^ on marine natural products exhibiting bone metabolism activity, we found that only 36 MNPs exhibited inhibitory effects on osteoclastogenesis, with only 14 MNPs demonstrating the ability to induce osteogenic differentiation. However, due to the limited yield, low bioavailability, and unclear mechanisms of these marine compounds, their clinical translation is limited, with no natural marine drugs available for clinical treatments. In this study, the active target compound VDC is investigated for the first time to assess its effects on osteoblast activity both in vivo and in vitro, along with elucidating its mechanism of action. The high yield (1.6 g/8 kg), strong osteogenic activity, definite molecular mechanisms, and therapeutic targets of VDC demonstrate its promising clinical application prospects. Additionally, VDC provides a marine molecular tool for combating bone metabolic diseases.

Wnt signaling regulates liver metabolism and regeneration, lung tissue repair and metabolism, hair follicle renewal, hematopoietic system development, and osteoblast maturation. In recent years, potential regulatory factors of the Wnt/β‐catenin signaling pathway have been identified, including SHN3, Twa1, FOXKs, ICAT, and Kdm2a/b. In osteoblasts, SHN3 plays various roles in regulating bone homeostasis by inhibiting osteoblast activity. These findings suggest that traditional osteoblast‐targeted therapies could be effective for treating low bone mass diseases and highlight the Wnt/SHN3 pathway as a promising target for inducing therapeutic bone metabolism responses. Notably, we used *de novo* sequencing to predict the large protein structure of SHN3 and determined the binding mode and energy between VDC and SHN3, providing a promising strategy for future research on molecular inhibitors of this protein. In vitro experiments of SHN3 deficiency have demonstrated that SHN3 is a key target for VDC‐induced osteoblasts, marking the first study to identify a natural small molecule ligand for SHN3.

However, this study was characterized by several limitations. First, there is potential to further optimize the material design. For example, engineering modifications to the NVs could help regulate the osteogenic microenvironment, or functional groups on VDC could be further modified to enhance its pharmacological efficacy. This approach could potentially inhibit osteoclast formation while simultaneously promoting osteogenic activity, achieving a double effect.^[^ ^29^ ^]^ However, it may introduce challenges associated with drug loading, release, as well as the potential loss of VDC active groups. Therefore, for future translational research, a direct NVs formulation is advised, with the VDC providing a promising foundation for developing drugs for bone diseases. Second, due to the unavailability of a SHN3 antibody for western blotting, our investigation on the impact of VDC on SHN3 protein was limited. This limitation arises from the significant size of SHN3, consisting of 2348 amino acids. Additionally, the low expression levels of SHN3 hindered the acquisition and purification of the protein in substantial quantities. Consequently, the 3D structure of SHN3 remains unclear. While we have attempted to predict the structure of SHN3, our findings necessitate further experimental evidence to validate, such as cryoelectron microscopy. Before assessing the SHN3 protein, procurement of a substantial quantity of its expression remains a significant challenge. Our investigation reveals that XCL‐5 can significantly promote the mRNA transcription of *SHN3* (Figure S1, Supporting Information), presenting a promising avenue for further exploration. Finally, the conventional tail vein injection method used in OIM heightens the risk of spontaneous fractures. Specifically, this risk can be mitigated by using anesthetic induction, or delivering modified particles through the nasal‐brain pathway.

Collectively, the quinolinone alkaloid VDC, obtained from deep‐sea‐derived fungus, significantly enhances osteogenic differentiation through the Wnt/SHN3 signaling pathway. It provides potential therapeutic benefits for osteoporosis treatment and fracture healing. Through this study, we also engineered SDSSD‐modified osteoblast membrane NVs containing VDC for targeted drug delivery to osteoblasts, offering a potential treatment option for OI. Our findings highlight the potential of using VDC to target the Wnt/SHN3 pathway for treating osteoporosis, fractures, and OI in clinical settings.

## 4 Experimental Section

### Materials

Alpha‐modified minimal essential medium (α‐MEM), fetal bovine serum (FBS), and penicillin/streptomycin were procured from Gibco, while β‐glycerophosphate (S0942), DMSO (34 869), Alizarin Red S (A5533), Oil Red O (1320‐06‐5) Alcian blue (33864‐99‐2) were obtained from Sigma. Toluidine blue (6586‐04‐5) was acquired from Macklin. Na_2_CO_3_ (497‐19‐8), NaHCO_3_ (144‐55‐8), and MgCl_2_ (7791‐18‐6) were purchased from Sinopharm Chemical Reagent Co., Ltd. Ascorbic acid (60374ES60) was sourced from Yeasen. Vazyme's HiScript II Q RT SuperMix (R22301), trizol (R40101AA), and ChamQ Universal SYBR qPCR Master Mix (Q71102) were gained from Vazyme for use in the study. The antibodies β‐Catenin (15B8, 1:1000), GSK‐3β (D5C5Z, 1:1000), phospho‐GSK‐3β (Ser9) (D3A4, 1:1000), NF‐κB (D14E12, 1:1000), ERK1/2 (137F5, 1:1000), phospho‐ERK1/2 (D13.14.4E, 1:2000) and GAPDH (D4C6R, 1:1000) were acquired from Cell Signaling Technology. The SHN3 antibody (HPA005728, 1:200) was purchased from Merck, while the Slit3 antibody (AF3629, 1:400) was obtained from R&D. Cell Counting Kit‐8 (CCK‐8, CT01A) was supplied by Cellcook. The primer sequences implemented in the qPCR were furnished by Origene. The organic reagents, such as chloroform, isopropanol, ethanol, and others, were all obtained from Sinopharm.

### Cell and Animals

The MC3T3‐E1 cell line (clone 4; CRL‐2593) was derived from the American Type Culture Collection, Rockville, MD, USA. Three‐ to six‐week‐old C57BL/6J mice were used to obtain BMSCs and bone marrow monocytes (BMMs). MC3T3‐E1 cells and BMSCs were plated in a normal culture medium (α‐MEM, 10% FBS, and 1% penicillin/streptomycin) and incubated at 37 °C in a 5% CO_2_ incubator. BMMs were cultured in a normal culture medium with 25 ng mL^−1^ M‐CSF at 37 °C and 5% CO_2_. Passage‐2 BMSCs, passage‐0 BMMs, and passage‐5 MC3T3‐E1 cells were used in this experiment.

The C57BL/6J mice were obtained from Gempharmatech Co., Ltd. *Col1a2* ^oim/oim^ mice were obtained from the Jaxson Laboratory (B6C3Fe *a/a*‐*Col1a2^oim^*/J, Stock No: 0 01815, Bar Harbor, ME, USA). *SHN3 ^−/−^* mice were described in the previous study.^[^ ^9^ ^]^ All mice were housed in up to five per cage under a 12 h light‐dark cycle with chow ad libitum in the laboratory animal center at Xiamen University. All mouse experiments were handled according to the protocols approved by the Institutional Animal Care and Use Committee of Xiamen University Laboratory Animal Center.

### Source and Identification of VDC

All 251 compounds from deep‐sea‐derived fungi were provided by professor Xianwen Yang. VDC was isolated from the deep‐sea‐derived fungus *Penicillium solitum* MCCC 3A00215. It was obtained as a white amorphous powder and identified by comparison of NMR and HRESIMS data with literature references. HRESIMS m/z 252.0744 [M − H]^−^; ^1^H‐NMR (400 MHz, *d*_6_‐DMSO) *δ*_H_ (ppm): 6.71 (2H, m), 6.83 (1H, d, J = 7.4 Hz), 7.09 (1H, m), 7.24−7.38 (3H, m), 9.19 (1H, s), 9.56 (1H, s), 12.23 (1H, s); ^13^C‐NMR (100 MHz, *d*_6_‐DMSO) *δ*_C_ (ppm): 114.7, 115.3, 116.7, 120.4, 121.0, 122.2, 124.1, 124.5, 126.5, 129.5, 133.1, 135.0, 142.3, 157.4, and 158.4.

### Osteoblast Differentiation

Briefly, MC3T3‐E1 cells were seeded at a density of 5000 cells per well into 96‐well plates containing normal culture medium and allowed to adhere overnight. The next day, the culture medium was substituted with osteoblast differentiation medium (culture medium supplemented with 50 µg mL^−1^ of ascorbic acid and 5 mm β‐glycerophosphate). Under inducing conditions, the cells were treated with VDC (1, 2.5, 5, and 10 µm) for 5 days. The medium was replenished every 2 days. Then the cells were subjected to an incubation process with alarmar blue (Invitrogen, DAL1100) for a duration of 2–4 h at a 37 °C in a 5% CO_2_ incubator. The resulting solution was gauged at a wavelength of 570 nm. Subsequently, the cells were treated with a substrate solution containing phosphatase substrate (Sigma, 3333338‐18‐4), 0.5 m Na_2_CO_3_, 0.5 m NaHCO_3_, 1 m MgCl_2_, and incubated for 30 min before being measured again at a wavelength of 405 nm.

### Osteogenesis Mineralization

The BMSCs were cultivated at a concentration of 2 × 10^4^ cells per well in 96‐well plates, and the MC3T3‐E1 cells were cultivated at a concentration of 5000 cells per well in 96‐well plates. Under osteogenic induction conditions, the cells were treated with VDC (1, 2.5, 5, 10, 15, and 20 µm). The differentiation medium was replenished every 48 h for 14 days for BMSCs, and 21 days for MC3T3‐E1 cells. The calcification conditions in cultures were evaluated via Alizarin Red staining. The cells were fixed in 4% neutral‐buffered formalin for 30 min, followed by incubation with 2% Alizarin Red S for 2 min. To quantify the results, 10% acetic acid was added to each well and the absorbance was measured using a spectrophotometer (BioTek Epoch2) at 405 nm.

### Adipogenic Differentiation

The BMSCs were cultivated at a concentration of 6 × 10^4^ cells per well in 96‐well plates, a utilizing conventional culture medium comprised of α‐MEM, 10% FBS, and 1% penicillin/streptomycin. Adipocyte differentiation was stimulated subsequent to the growth of cells reaching confluence through the substitution of culture medium enriched with 5 µm dexamethasone (Solarbio, D8041), 0.5 mm 3‐isobutyl‐1‐methylxanthine (Sigma, 28822‐58‐4), 0.5 µg mL^−1^ insulin transferrin‐selenium solution (OriCella, ITSS‐10201), 1 µm rosiglitazone (Sigma, 122320‐73‐4), and incubating for 6 days (medium was replaced every day). Under the induction conditions, the cells were treated with VDC (1, 5, and 10 µm). The cells were washed with PBS and subsequently fixed with 4% formaldehyde. The stained cells were then incubated with filtered Oil Red O/60% isopropanol solution at room temperature for 1 h. Adipocytes that showed red staining were captured using light microscopy (Olympus CKX3‐SLP, Japan).

### Chondrogenic Differentiation

The BMSCs were seeded at a density of 6 × 10^5^ cells per well in 12‐well plates. After 24 h, the cell was allowed to completely immerse in the chondrogenic differentiation medium. This solution was enriched with 1% insulin transferrin‐selenium solution, 10 ng mL^−1^ TGF‐β3 (MedChemExpress, HY‐P7120), 100 nm dexamethasone, 40 µg mL^−1^ proline (Sigma‐Aldrich, 147‐85‐3), 50 µg mL^−1^ l‐ascorbic acid 2‐phosphate (Yeasen biotech, 60374ES60). Under the induction conditions, the cells were treated with VDC (1, 5, and 10 µm) for a duration of ≈21 days. Cells were fixed with 4% neutral formaldehyde for 30 min after two PBS washes, then stained with 1% Alcian blue for 30 min. The chondrogenic staining was observed and imaged under a microscope, and the differentiation degree was quantified using Image J.

### qPCR

Total RNA was isolated using the trizol method (Vazyme, R40101AA) followed by reverse transcription using Vazyme's HiScript II Q RT SuperMix (R22301) for qPCR in accordance with the instructions provided by the manufacturer. Gene expression levels were detected through the utilization of ChamQ Universal SYBR qPCR Master Mix (Vazyme, Q71102), with six replications performed for each experiment. The comparative cycle threshold method was employed to analyze gene expression, with HPRT as the internal control. The 2^−△△CT^ method was used to normalize the data. Specific primers with SYBR Green (based on the mouse sequences) are listed in Table S1 (Supporting Information).

### RNA‐Sequencing and Data Processing

Pre‐osteoblasts MC3T3‐E1 cells were plated into 6‐well plates at 1 × 10^5^ cells per well. Under the induction conditions of 50 µg mL^−1^ ascorbic acid and 5 mm β‐glycerophosphate, MC3T3‐E1 cells were treated with or without VDC for 3, 5, and 8 days. The medium was refreshed every 2 days. 0.1% DMSO was used as a control. As mentioned previously,^[^ ^28c^ ^]^ the total RNA was extracted using the TRIzol reagent (Invitrogen, CA, USA) according to the manufacturer's protocol. Differential expression analysis was performed using the DESeq2 Q value <0.05 and fold change >2 or <0.5 was set as the threshold for DEGs. GO and KEGG pathway enrichment analysis of DEGs was performed to screen the significantly enriched term using R (v 3.2.0), respectively. The PPI network was created by the STRING database (https://cn.string‐db.org/) and visualized using Cytoscape v3.7.0 or v3.10.1 software.

### Western Blotting

As previously,^[^ ^28c^ ^]^ MC3T3‐E1 cells were plated at a density of 10 × 10^5^ cells per well into 6‐well plates. Under induction conditions with 50 µg mL^−1^ ascorbic acid and 5 mm β‐glycerophosphate, MC3T3‐E1 cells were cultured with VDC (1, 5, and 10 µm) or 0.1% DMSO (control group) for 5 days. Cells were lysed with RIPA buffer (Solarbic, R0010) supplemented with protease inhibitor cocktail (MCE, HY‐K0010) and phosphatase Inhibitor Cocktail II (MCE, HY‐K0022). The lysate was mixed with SDS sample buffer and subsequently heated to 95 °C for 5 min. Equal amounts of protein were resolved by SDS‐PAGE (Sangon Biotech, 151‐21‐3) and transferred onto a PVDF membrane (Millipore, IPVH00010). The membrane was blocked with 5% milk and then incubated with primary antibodies overnight at 4 °C. The next day, secondary antibodies were applied and the immunoblots were visualized using chemiluminescence. The results were measured using ImageJ to quantify the intensity of the target protein and GAPDH bands.

### Prediction of SHN3 Protein Structure and Molecular Docking

ITASSER generated the predicted 3D structure of SHN3 using amino acids 327–1183 (NP_0 011 21186.1). The model quality was evaluated with Laplace plots from the SAVES server. The protonation state of all the compounds was set at pH 7.4, and the compounds were expanded to 3D structures using Open Babel.^[^ ^30^ ^]^ AutoDock Tools (ADT) were applied to prepare and parametrize the receptor protein (SHN3) and VDC. The docking grid documents were generated by AutoGrid of the sitemap, and AutoDock Vina (1.2.0) was used for docking simulation.^[^ ^31^ ^]^ The optimal pose was selected to analyze interaction. Finally, the protein‐ligand interaction figure was generated by PyMOL and Schrodinger software (Schrödinger Maestro, New York, NY, USA. Version 11.1.011, MM share Version 3.7.011, Release 2017‐1, Platform Windows‐x64). The SHN3 protein was represented as a slate cartoon model, ligand was shown as a green stick, and their binding sites were shown as magenta stick structures. Nonpolar hydrogen atoms are omitted. The hydrogen bond, ionic interactions, and hydrophobic interactions were depicted as yellow, magenta, and green dashed lines, respectively.

### Molecular Dynamics (MD) Simulations

The molecular dynamics simulations were conducted using Desmond/Maestro (Shaw, D. E. Desmond molecular dynamics system, version 2022.1. Research, New York, NY).^[^ ^32^ ^]^ TIP3P water molecules were added to the systems, which were then neutralized by 0.15 m NaCl solution. *Following system minimization and relaxation*, the production simulation was performed for 100 ns *under isothermal‐isobaric (NPT) conditions at 300 K and 1* *bar*. Trajectory coordinates were recorded every 100 ps. The *MD* analysis *was carried out using* Simulation Interaction Diagram from Desmond.

### Immunofluorescence

BMSCs were grown on glass‐bottom cell culture dishes (NEST, 801001‐1, 20 mm, TC) and treated with VDC for 3, 5, and 8 days. The BMSCs were rinsed with ice‐cold PBS twice and fixed with 4% paraformaldehyde for 30 min, followed by permeabilization with 0.3% Triton X‐100 in PBS for 15 min. The cells were blocked with 5% bovine serum albumin for 60 min. Then they were incubated with β‐catenin antibody (1:3000 dilution) and OCN antibody conjugated with mouse Alexa Fluor 597 and (1:200 dilution) conjugated with rabbit Alexa Fluor 488 in 5% BSA for 4 degrees overnight, followed by staining with DAPI (0.1 µg mL^−1^, 1:10 000) for 15 min. The cells were washed with PBS three times and viewed on a fluorescence microscope (Olympus, APEXVIEW APX100, Japan)

For immunofluorescence of bone tissue, freshly dissected bone dissected and soft tissues from mice were collected and immediately fixed in ice‐cold 4% paraformaldehyde solution overnight. Decalcification was especially carried out with 0.5 m EDTA at 4 °C with constant shaking for bone samples from mice. All samples were embedded in OCT or CPT (Sakura) and cut into 40‐um‐thick sagittal sections using a cryostat (Leica). Immunofluorescence staining and analysis were performed according to a published protocol.^[^ ^9^ ^]^

### Synthesis of the Bone‐Targeting Peptide

The bone‐targeting peptide SDSSD (Ser‐Asp‐Ser‐Ser‐Asp, 98% purity as determined by HPLC) was synthesized by Wansheng Haotian Biological Technology (Shanghai, China), and 1,2‐distearoyl‐sn‐glycero‐3‐phosphoethanolamine‐N‐[methoxy(polyethyleneglycol)‐2000]‐NHS (DSPE‐PEG2000‐NHS, 98% purity as determined by HPLC) was synthesized by Xi'an Ruixi Biological Technology Company (Xi'an, China). Both compounds were individually dissolved in 5 mm HEPES buffer at 1 mg mL^−1^. The DSPE‐PEG2000‐NHS was then reacted with the peptide in a molar ratio of 1:1 at room temperature for 24 h to produce DSPE‐PEG2000‐SDSSD. The mixture was dialyzed against distilled water using a dialysis bag (molecular weight cutoff of 3000 Da) for 48h. Finally, the dialyzed solution was freeze‐dried and the DSPE‐PEG2000‐SDSSD powder was stored at −20 °C.^[^ ^33^ ^]^

### Preparation and Characterization of BT‐NVs

First, MC3T3‐E1 cells were collected to prepare NVs. The cell membranes derived from MC3T3‐E1 cells were isolated by multistep density gradient centrifuge (2000 rpm 10 min, 5000 rpm 10 min, and 15 000 rpm 60 min), following sonication.^[^ ^34^ ^]^ Then the collected membranes were resuspended in physiological saline and extruded through 1, 0.45, 0.22, and 0.1 µm polycarbonate porous membrane filters. The NVs were then resuspended and further purified by washing with physiological saline, and determined using the BCA (Pierce BCA Protein Assay Kit; Thermo).

The bone‐targeted peptide was mixed with NVs at a ratio of 1:1 (protein weight), and incubated overnight at 4 °C. To remove uncombined DSPE‐PEG‐SDSSD, the mixture was washed with physiological saline via centrifugation and resuspended. The NVs and BT‐NVs were then characterized and analyzed using TEM (Tecnai G2 Spirit BioTwin; Tools for NanoTech FEI), DLS (Zetasizer Nano ZS‐90; Malvern Instruments), and ZETA (Zetasizer Nano ZS‐90; Malvern Instruments).

### Drug Loading

In brief, for the preparation of NVs‐VDC (BT‐NVs‐VDC), 100 µg (protein weight) of NVs (BT‐NVs) and 100 µg of VDC were mixed, sonicated with the following settings: 20% amplitude, 6 cycles of 30 s on/off for three minutes with a 2 min cooling period between each cycle. After sonication, the mixture was left for 60 min to allow for membrane recovery, followed by centrifugation to remove free VDC. The drug loading efficiency (DLE) of VDC was quantified using HPLC (Agilent 1260; Agilent).^[^ ^35^ ^]^

(1) $$\mathrm{DLE}\;\left(\%\right)=\frac{\mathrm{Weight}\;\mathrm{of}\;\mathrm{Drug}\;\mathrm{loaded}}{\mathrm{Weight}\;\mathrm{of}\;\mathrm{drug}\;\mathrm{loaded}+\mathrm{Weight}\;\mathrm{of}\;\mathrm{nanovesicles}}\times \;100\%$$

### Drug Release

BT‐NVs‐VDC were transferred into dialysis bags (molecular weight cutoff of 3000 Da) and then immersed in 100 mL of PBS (pH 7.4) at room temperature. Samples of 200 µL were taken at different time periods and stored at 4 °C. At the same time, the corresponding volume of PBS was added for volume supplement. Released VDC was detected by HPLC (Agilent 1260; Agilent).

### Cellular Uptake of NVs (BT‐NVs) In Vitro

Cellular uptake was assessed using flow cytometry and confocal laser scanning microscopy (CLSM). MC3T3‐E1 cells were seeded into observation dishes at a density of 1 × 10^4^ cells per dish. After the cells reached 80% confluence, the medium was exchanged for α‐MEM containing NVs or BT‐NVs labelled with DiR iodide and incubated at 37 °C. After different incubation periods, the supernatant was carefully removed for flow cytometry (Attune Nxt; Thermo).

For CLSM analysis, the cells were gently washed three times with PBS and fixed with 4% paraformaldehyde for 10 min. Then the cells were washed another three times, stained with 4′,6‐diamidino‐2‐phenylindole (DAPI) for 30 min and ultimately observed under CLSM (Leica TCS SP8 DLS; Leica Microsystems Ltd).

### Biodistribution of NVs (BT‐NVs) In Vivo

Each mouse was injected intravenously with DiR‐labeled NVs (BT‐NVs) at a dose of 100 µg. After different time intervals, the mice were euthanized. The major organs (heart, liver, spleen, lung, kidney, and femur) were isolated to analyze the fluorescence intensity using IVIS Spectrum (PerkinElmer, USA).

### Ovariectomised (OVX)‐Induced Osteoporosis Mouse Model

Bilateral ovariectomy was performed to induce systemic osteoporosis in the mice. Female 10‐weeks old C57BL/6J mice obtained from Gempharmatech Co., Ltd, were used to establish an OVX mice model. The mice were then randomly divided into three groups (*n* = 8): sham (without surgery); OVX; OVX+5 mg kg^−1^ VDC groups. The sham or OVX group received intravenous injections of physiological saline, while the OVX+VDC group were administered 5 mg kg^−1^ VDC every day. All the mice were euthanized after 6 weeks of controlled feeding, with 20 mg kg^−1^ Calcein (Sigma, 154071‐48‐4) administered subcutaneously to the mice at ‐8 and ‐1 days before euthanization. Bones were harvested at the indicated time points for histological and µCT analyses.

### Fracture Model

Eight‐week‐old C57BL/6J female mice were randomly divided into two groups. Closed transverse diaphyseal fractures of the right femur were generated as previously described.^[^ ^9^ ^]^ A small electric saw was used to excise the middle part of the mice femur, resulting in linear fractures of the femur. A sterile 1 mL needle was inserted into the knee joint of the mouse. The broken end of the mouse fracture was connected while the needle was removed with a small tool saw, and the fracture was stabilized using the needle. One day post‐surgery, phenol red‐free Matrigel (Corning) was mixed with VDC (0.1 mg per 50 µL) for the VDC group treatment and applied immediately directly around the fracture site using an insulin syringe. Then the mice were treated with PBS (control group) or VDC (5 mg kg^−1^, I.V. three times per week, VDC group). All mice were euthanized on days 14 post‐fracture, with the fractured bones harvested at the indicated times for histological and µCT analyses.

### Osteogenesis Imperfecta Model

Four‐week‐old male *Col1a2* ^oim/oim^ mice were randomly assigned into five groups (OIM+PBS, OIM+freeVDC, OIM+BT‐NVs, OIM+NVs‐VDC, and OIM+BT‐NVs‐VDC) comprising six mice each. The control group consisted of 4‐week‐old male C57BL/6J mice. The mice received intravenous tail injections every 2 days for 4 weeks continuously. The samples were then collected, including serum, organs, and femurs. µCT was used to scan the femurs and measure the bone parameters.

### µ‐CT and Bone Histomorphometry Analysis

The right femurs were immobilized by fixation with 4% paraformaldehyde for 24 h, and then treated with 75% ethanol for further scanning. Cortical and trabecular bone morphometric parameters of the femur were evaluated using a high‐resolution µCT (SkyScan, United States) in the distal femoral bone. The micro‐CT scans were reconstructed with the NRecon program, and analyzed using the CTAn program as described.^[^ ^9^ ^]^

### Bone Tissue Staining

In brief, Bone tissues were fixed in ice‐cold 4% paraformaldehyde overnight, decalcified with 0.5 M EDTA at 4 °C with constant shaking, and then embedded in resin or paraffin wax. After deparaffinization and hydration, the tissues were stained using Von Kossa, Toluidine blue, Tartrate resistant acid phosphatase, and Safranin O/Fast Green methods, following a published protocol.^[^ ^9^ ^]^ Histomorphometric analysis was conducted using the Osteomeasure System Histomorphometric (OsteoMetrics, Atlanta, USA).

### Statistical Analysis

Data were analyzed using the GraphPad Prism 9.5. The figures and/or legends contained statistical tests, n values, replicate experiments, and *p* values. Two‐tailed Student's t‐test was used to assess significance between the two groups, while one‐way ANOVA with Tukey's post‐hoc test was employed to evaluate significance among multiple groups. Statistical significance was defined as a *p* value < 0.05. Error bars indicate mean ± SD. Ns: not significant, *p < 0.05, **p < 0.01, ***p < 0.001, and ****p < 0.0001 represented statistical significance.

### Ethical Approval Statement

The animal study was reviewed and approved by The Laboratory Animal Management and Ethics Committee of Xiamen University (XMULAC20190084).

## Conflict of Interest

The authors declare no conflict of interest.

## Author Contributions

X.C.L., Y.S.H., and Y.Y.T. contributed equally to this work. X.C.L., Y.Y.T., and Y.S.H. performed conceptualization. X.C.L., Y.Y.T., Y.S.H., S.B.H., and J.P.X. performed methodology. X.C.L. and Y.S.H. performed investigation. X.C.L., Y.Y.T., Y.S.H., L.L.J., Z.Z.B., and L.N. performed visualization. Y.X.W. and X.R. performed supervision. X.C.L. and Y.S.H. performed wrote the original draft. X.C.L., Y.Y.T., Y.S.H., G.M.B., Y.X.W., and X.R. performed wrote, reviewed, edited the draft.

## Supporting information



Supporting Information

## Data Availability

The data that support the findings of this study are available from the corresponding author upon reasonable request.
