# Supplementary material for: Viridicatol from the Deep‐Sea‐Derived Fungus Alleviates Bone Loss by Targeting the Wnt/SHN3 Pathway
Source: Adv Sci (Weinh). 2025 Apr 7;12(21):2416140. doi: 10.1002/advs.202416140 (PMC12140338; doi:10.1002/advs.202416140)
Supplement: Supplementary file 1 — Supporting Information [file ADVS-12-2416140-s001.docx]

Supporting Information

Viridicatol from the Deep-Sea-Derived Fungus Alleviates Bone Loss by Targeting the Wnt/SHN3 Pathway

Chun-Lan Xie, Shang-Hui Ye, Yu-Ting Yue, Bao-Hong Shi, Jing-Ping Xu, Lian-Jie Li, Zheng-Biao Zou, Matthew B. Greenblatt, Na Li*, Xian-Wen Yang*, Ren Xu*

1. **Supplemental materials and methods**
   1. *Hematoxylin and Eosin (HE) staining*

The paraffin slices were dewaxed with xylene and then dehydrated. Then it was staining according to the Hematoxylin and Eosin Staining Kit (C0105, Beyotime Institute of Biology). The H&E staining sections were mounted with neutral resin and observed under microscope.

- 1. *Osteoclasts differentiation*

The BMMs were cultivated at a concentration of 5000 cells/well in 96-well plates. Under induction conditions of M-CSF (25 ng/mL) and RANKL (25 ng/mL), the cells were treated with VDC (0, 1, 5, 10 μM). The differentiation medium was replenished every 48 hours for the duration of 6 days. For tartrate-resistant acid phosphatase (TRAP) staining, cells were ﬁxed and stained to assess TRAP activity. The staining images were captured using light microscopy (Olympus CKX3-SLP, Japan). TRAP-positive multi-nucleated cells with 3 nuclei were counted as osteoclasts. The number of TRAP-positive cells per well relative to the control was calculated.

- 1. *Tube formation assay*

Endothelial cell culture and tube formation assay was described as previously^[9]^. HUVEC were purchased from Lonza (#C2517A, Lonza) and cultured according to the manufacturer’s specifications. The HUVEC cells were maintained in Endothelial Cell Growth Medium 2, containing 2% of FCS, in a humidified atmosphere at 37 °C in 5% CO_2_. Endothelial cell tube formation assay was set up in 96-well plates precoated that had been precoated with Matrigel (Corning). Following a 1 h serum starvation, HUVEC were seeded at a density of 1.5 × 10^4^ cells per well in conditioned medium (DMEM+ECG 50×) containing varying concentrations of the VDC (0, 1, 2.5, 5, 10 μM). After a 4-hour incubation at 37 °C, the number of tube branches in each well was observed using microscopy. Subsequently, the images were analyzed utilizing the angiogenesis analyzer plugin of the Fiji software.

1. **Supplemental figures and tables**


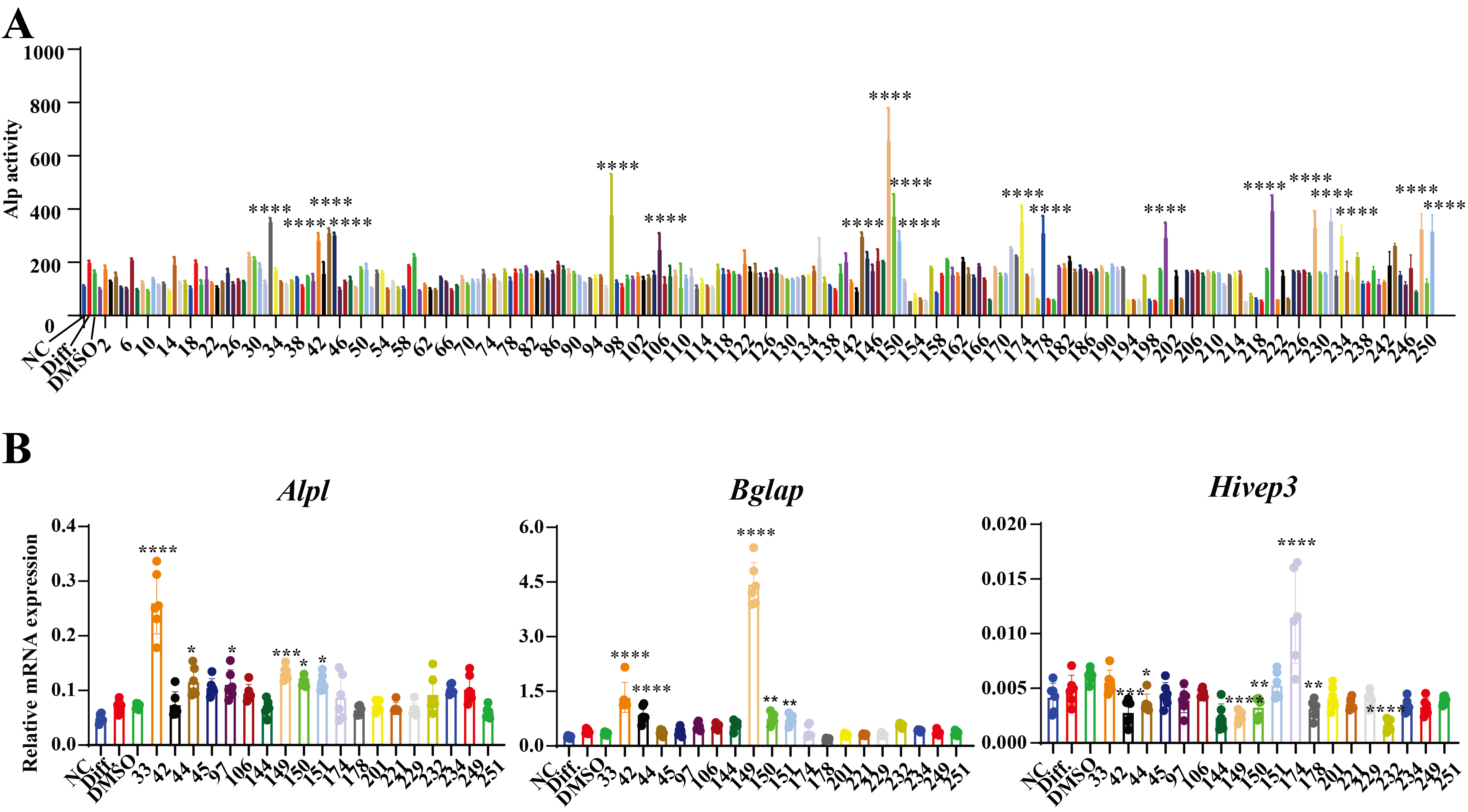


**Figure S1** Preliminary screening results demonstrating the effects of compounds from marine fungi on osteogenic differentiation. (**A**) Analysis of the Alp activity for the 251 marine natural compounds. The marine natural compounds (10 μM) were added to MC3T3-E1 cells for 5 days, under osteogenesis induction conditions (n = 3). All of compounds were separated by repeated column chromatography (CC) over ODS, silica gel, and Sephadex LH-20 and semi-preparative high performance liquid chromatography (HPLC), and identified by comparison of NMR and HRESIMS data with literature references. (**B**) qPCR analysis was performed to explore the effects of 19 potentially active compounds on the expression of osteogenic-specific genes *Alpl*, *Bglap*, and *Hivep3*. The 19 potentially active compounds (10 μM) were added to MC3T3-E1 cells for 5 days, under osteogenesis induction conditions, n=6. The normal culture medium, osteogenesis differentiation medium, and 0.1% DMSO served as the negative control group (NC), differentiation (Diff.), and solvent group (DMSO), respectively. Data represent mean ± SD, ns: not significant, **p* < 0.05, ***p* < 0.01, ****p* < 0.001, *****p* < 0.0001 vs the DMSO group by one-way ANOVA with Tukey’s post-hoc test.

**
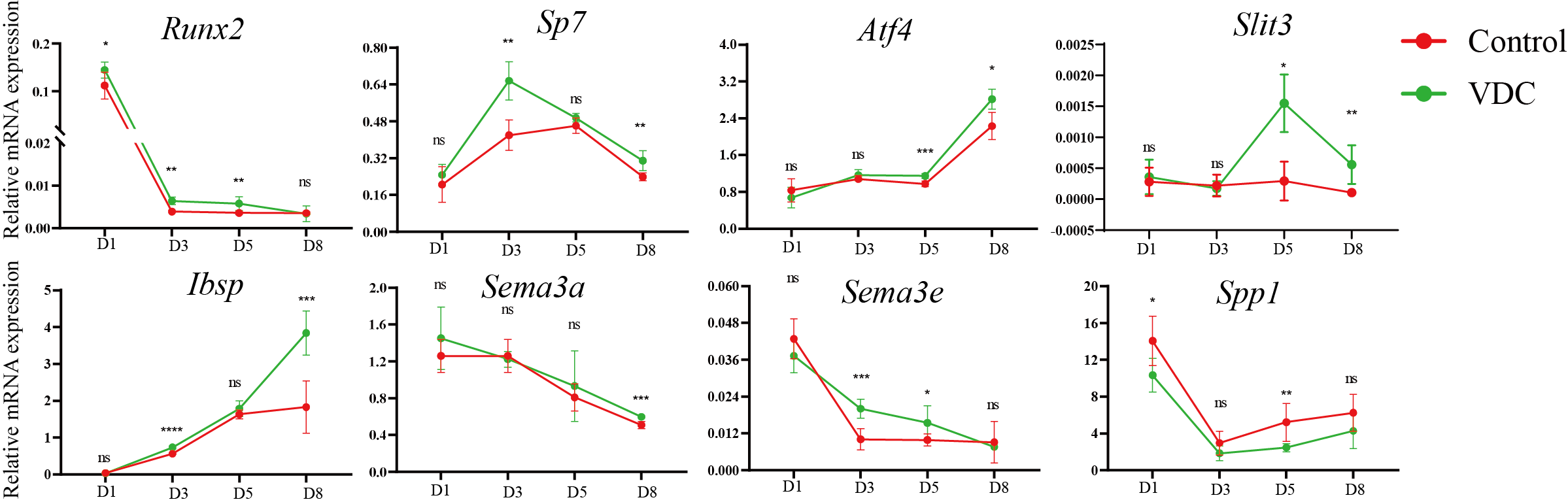
**

**Figure S2** The effect of VDC on the expression levels of osteogenic-specific gene among MC3T3-E1 cells by qPCR analysis. Under induction conditions, 5 μM VDC was added to MC3T3-E1 cells for different durations (D1, D3, D5, D8). (*n* = 6). 0.1% DMSO was the control group. Data represent mean ± SD, ns: not significant, **p* < 0.05, ***p* < 0.01, ****p* < 0.001, *****p* < 0.0001 by an unpaired two-tailed Student's *t*-test.


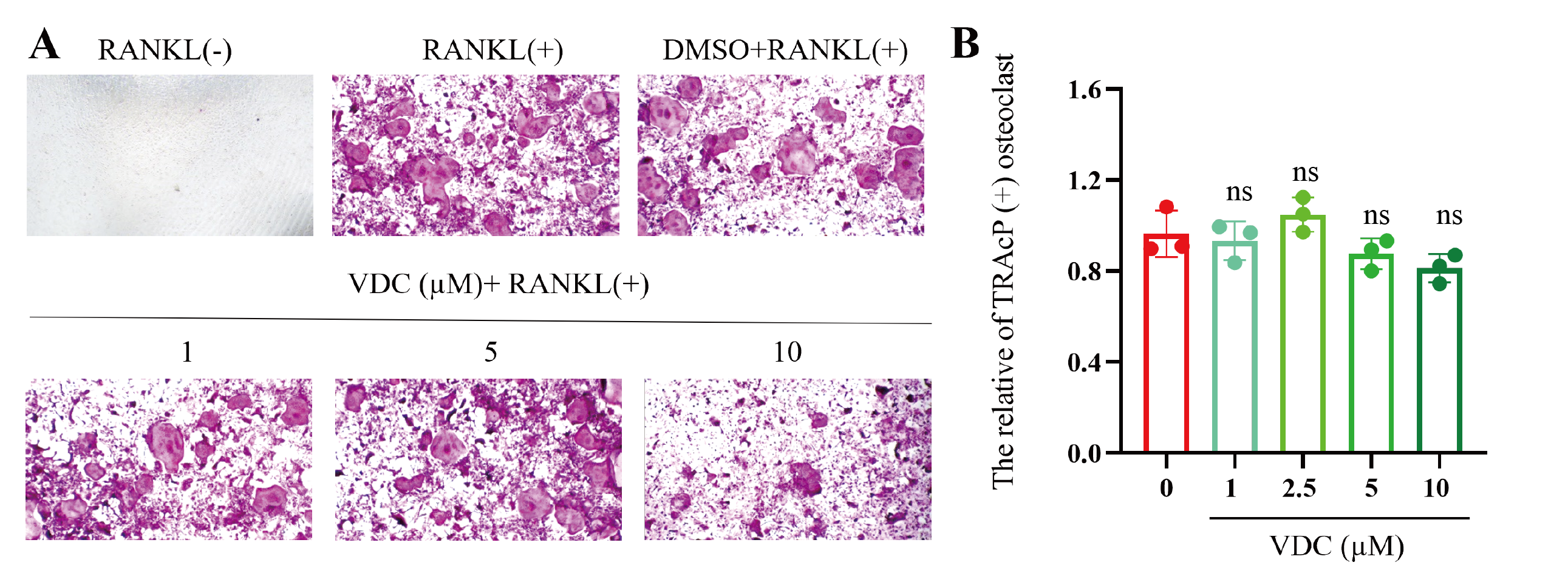


**Figure S3** The effects of VDC on osteoclasts at different doses. (A) Representative images of TRAP staining show BMMs treated with VDC during RANKL-induced osteoclastogenesis. (B) Quantification of TRAP-positive osteoclasts per well. Scale bar, 100 µm (n = 3). The bar graph is presented as mean ± SD. Scale bar, 200 µm. ns: not significant vs the 0 μM concentration group by one-way ANOVA with Tukey’s post-hoc test.


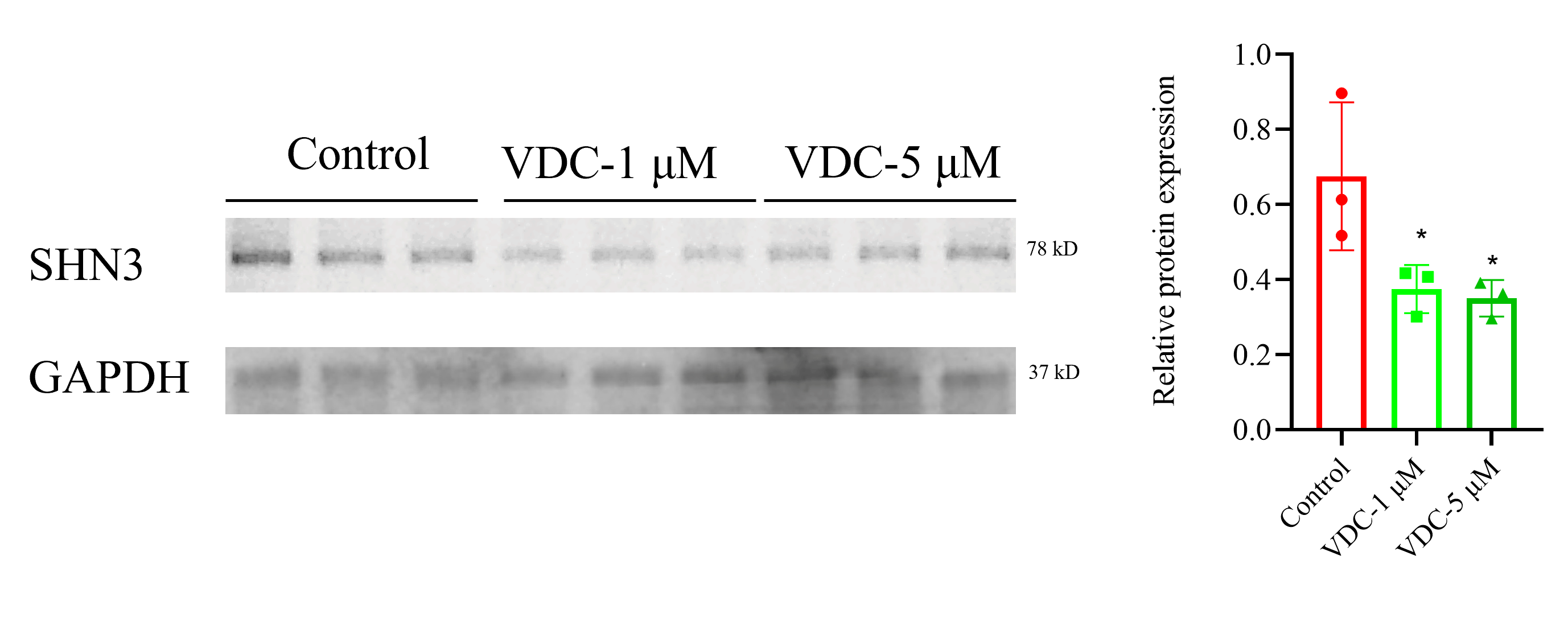


**Figure S4** VDC suppresses the expression of SHN3 protein levels. Representative western blot images showing the expression levels of SHN3, normalized to GAPDH expression. MC3T3-E1 cells were cultured under induction conditions (50 μg/mL ascorbic acid and 5 mM β-glycerophosphate) with VDC at concentrations of 1, 5 μM or with 0.1% DMSO as the solvent control group for 5 days. The control group (Control) was treated with 0.1% DMSO (n = 3). Data represent mean ± SD, **p* < 0.05 vs the Control group by one-way ANOVA with Tukey’s post-hoc test.


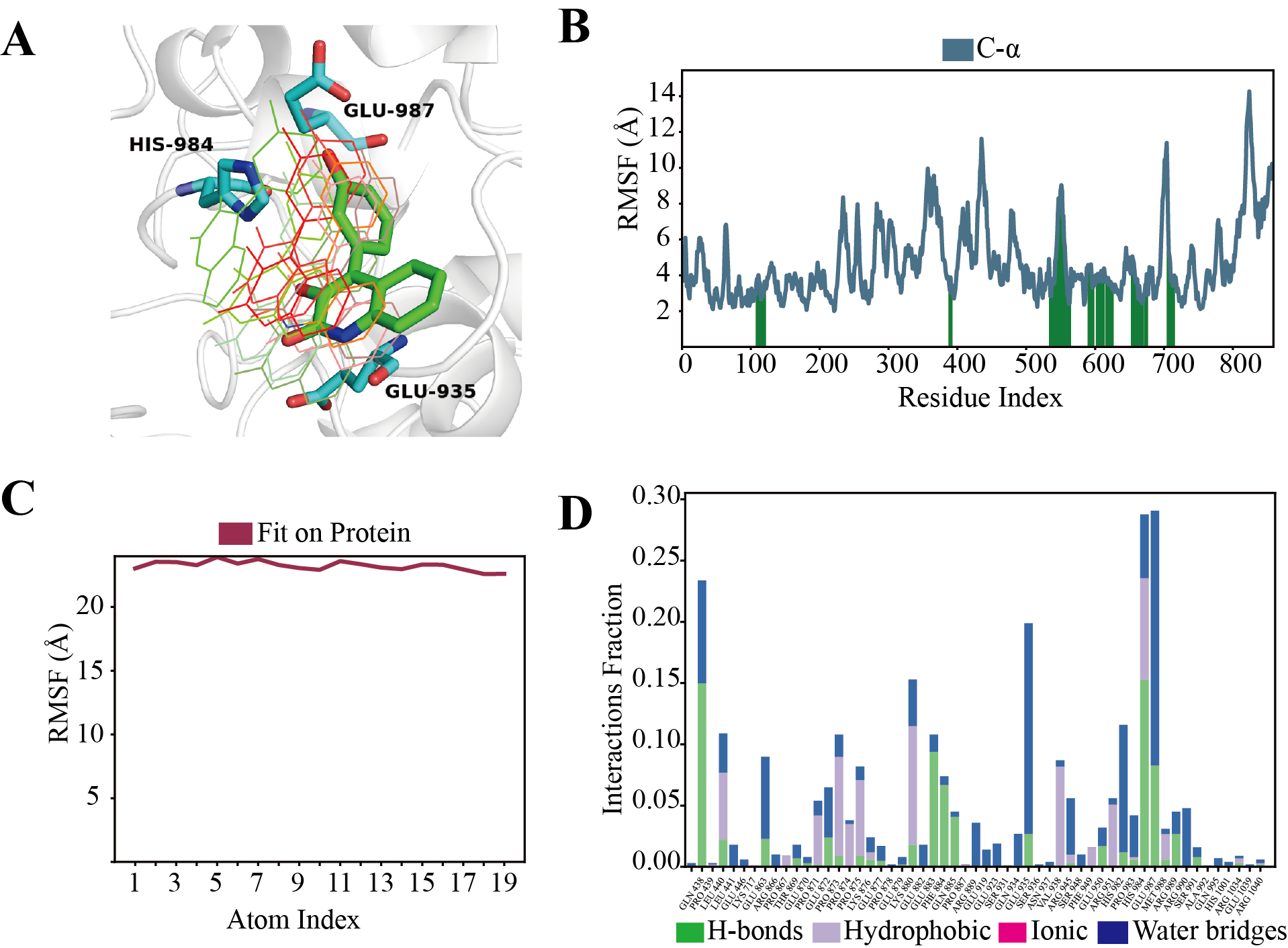


**Figure S5** Molecular dynamics simulations of VDC and SHN3. (**A**) VDC binding poses were obtained at intervals of 10 ns during the total simulation time of 100 ns. (**B**) The Root Mean Square Fluctuation (RMSF) is useful for characterizing local changes along the SHN3 protein chain. (**C**) The Ligand Root Mean Square Fluctuation (L-RMSF) is useful for characterizing changes in the VDC atom positions. (**D**) Interaction fractions for SHN3 and compound VDC in the 100 ns simulations. Protein interactions with compound VDC was monitored throughout the simulation and are characterized by hydrogen bonds (H-bonds; green); hydrophobic contacts (purple); ionic bonds (pink), and water bridges (blue). The interaction fraction describes how long interactions are maintained over the course of the trajectory snapshots. An interaction fraction of 1.0 suggests that this interaction is maintained in the simulation 100% of the time.


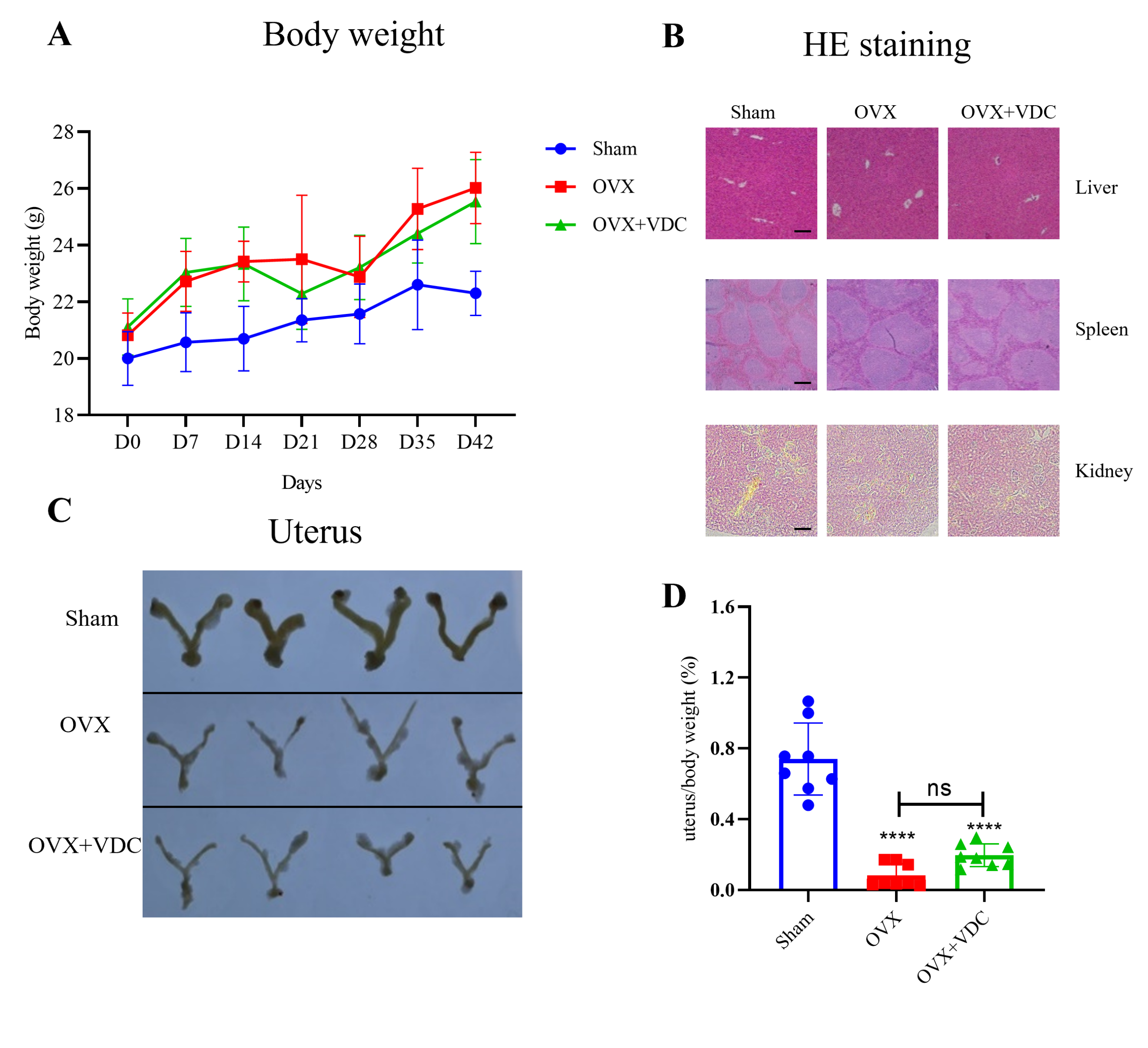


**Figure S6** Biosafety of VDC. (A) Tracking of body weights in different groups of mice (n = 8). (B) Representative H&E staining images of the main organs of mice (liver, spleen, and kidney). Scale bar, 100 μm. (C) Photograph of the uterus. (D). The uterus weights tracking of different groups mice (*n* = 8). Data represent mean ± SD, ns: not significant, *****p* < 0.0001 vs the Sham group by one-way ANOVA with Tukey’s post-hoc test.

**
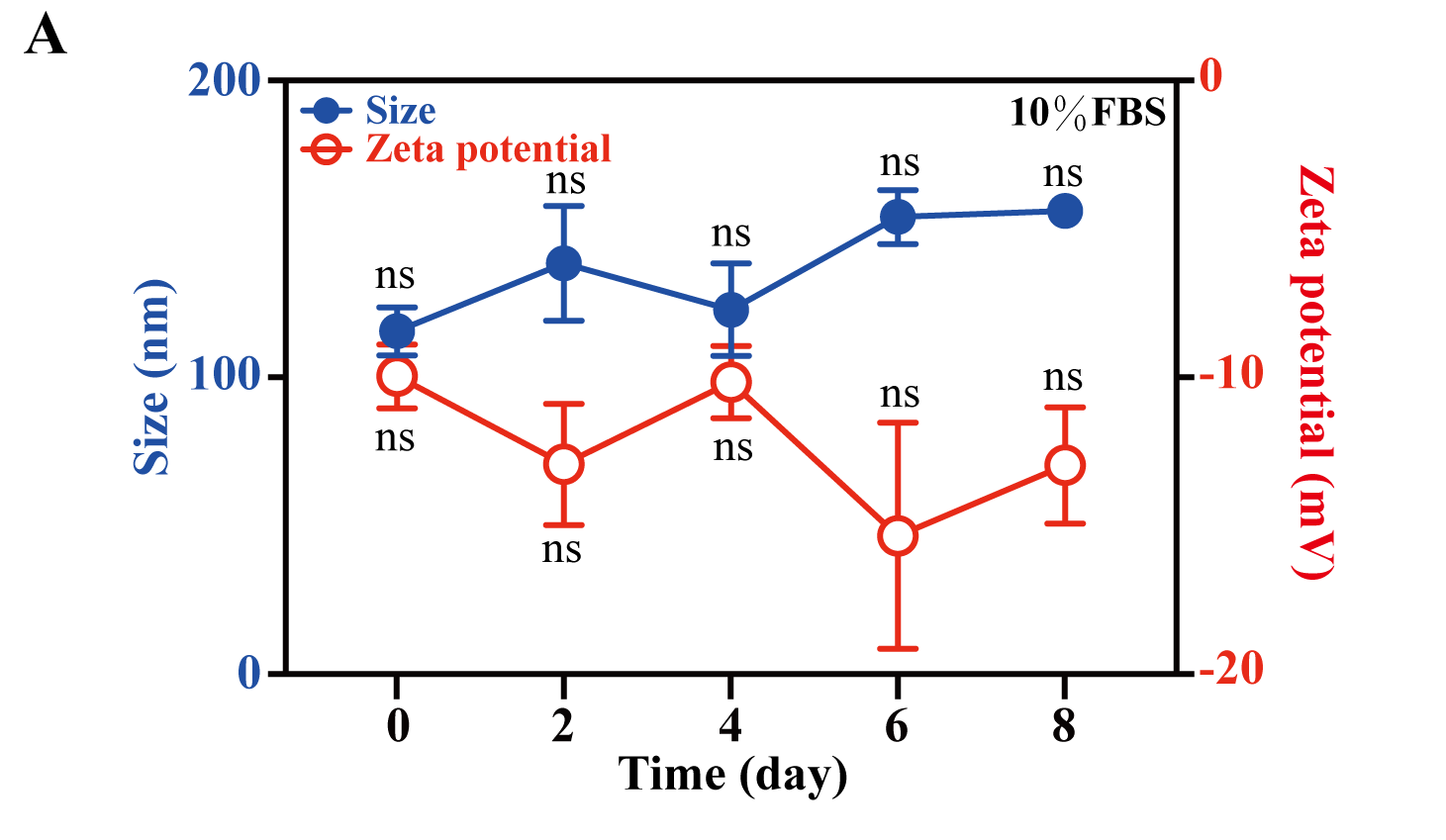
**

**Figure S7** Study of the stability of BT-NVs in 10% FBS over 8 days (n = 3). Data represent mean ± SD, ns: not significant vs the previous measurement by an unpaired two-tailed Student's *t*-test.


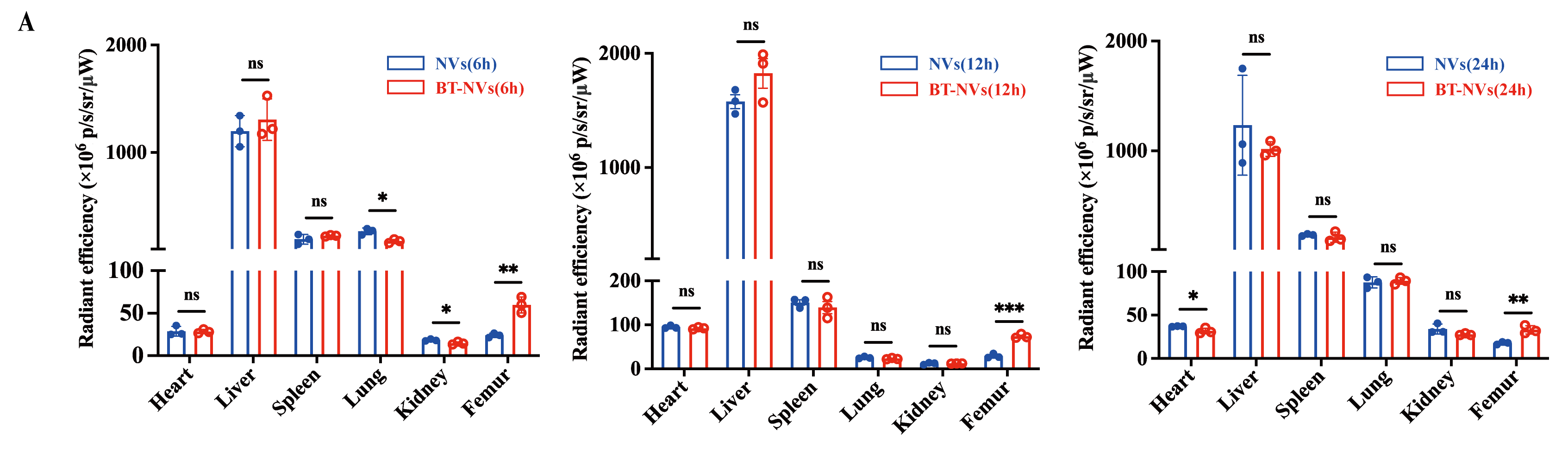


**Figure S8** (A) Radiant efficiency of DiR-labelled NVs or BT-NVs in different organs (heart, liver, spleen, lung, kidney, and femur) (n = 3). Data represent mean ± SD, ns: not significant, **p* < 0.05, ***p* < 0.01, ****p* < 0.001, *****p* < 0.0001 by an unpaired two-tailed Student's *t*-test.


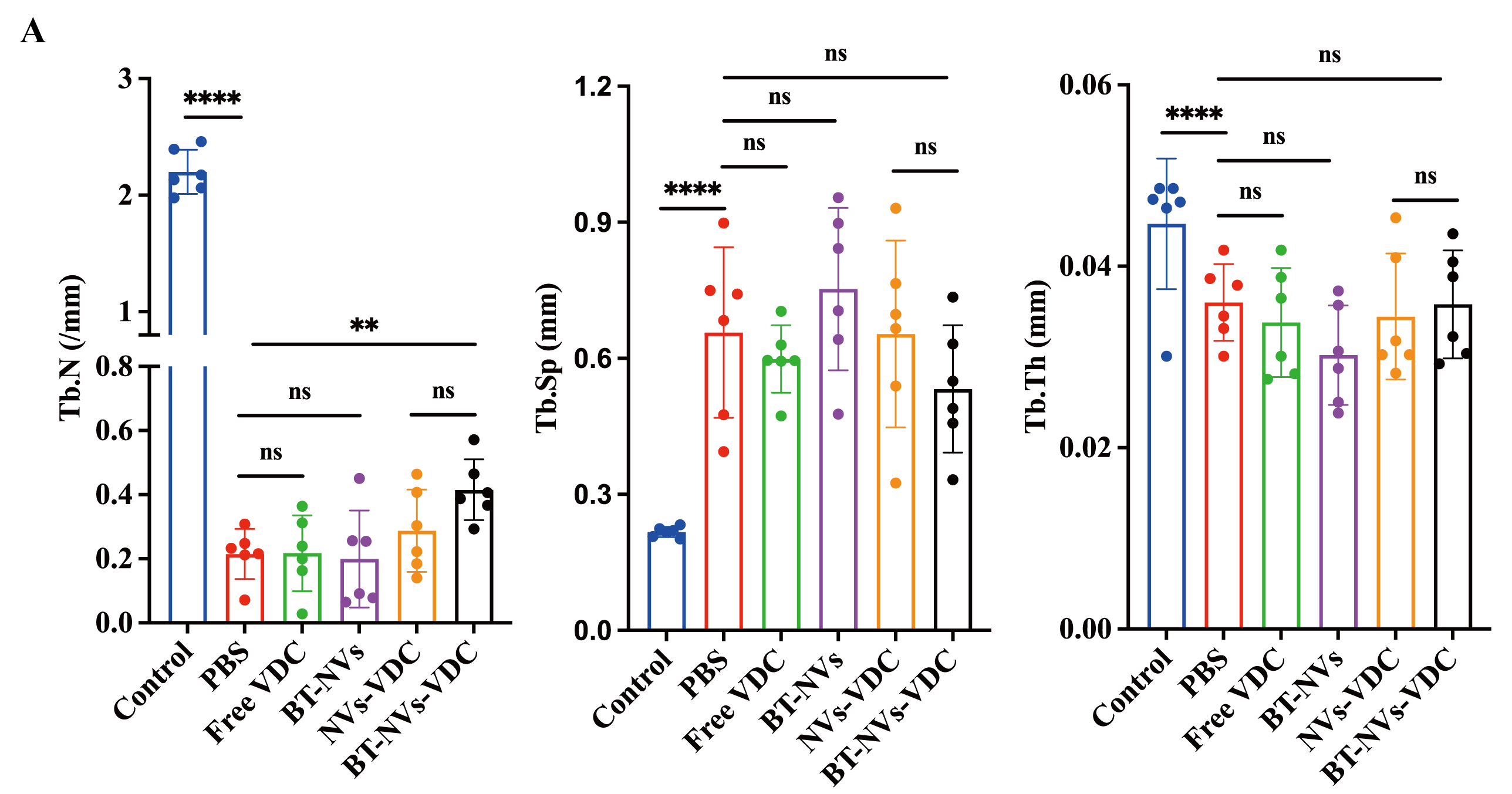


**Figure S9** Quantitative measurements of bone microstructure-related parameters: Tb.N, Tb.Sp, and Tb.Th (n = 6). Data represent mean ± SD, ns: not significant, **p* < 0.05, ***p* < 0.01, ****p* < 0.001, *****p* < 0.0001 by one-way ANOVA with Tukey’s post-hoc test.


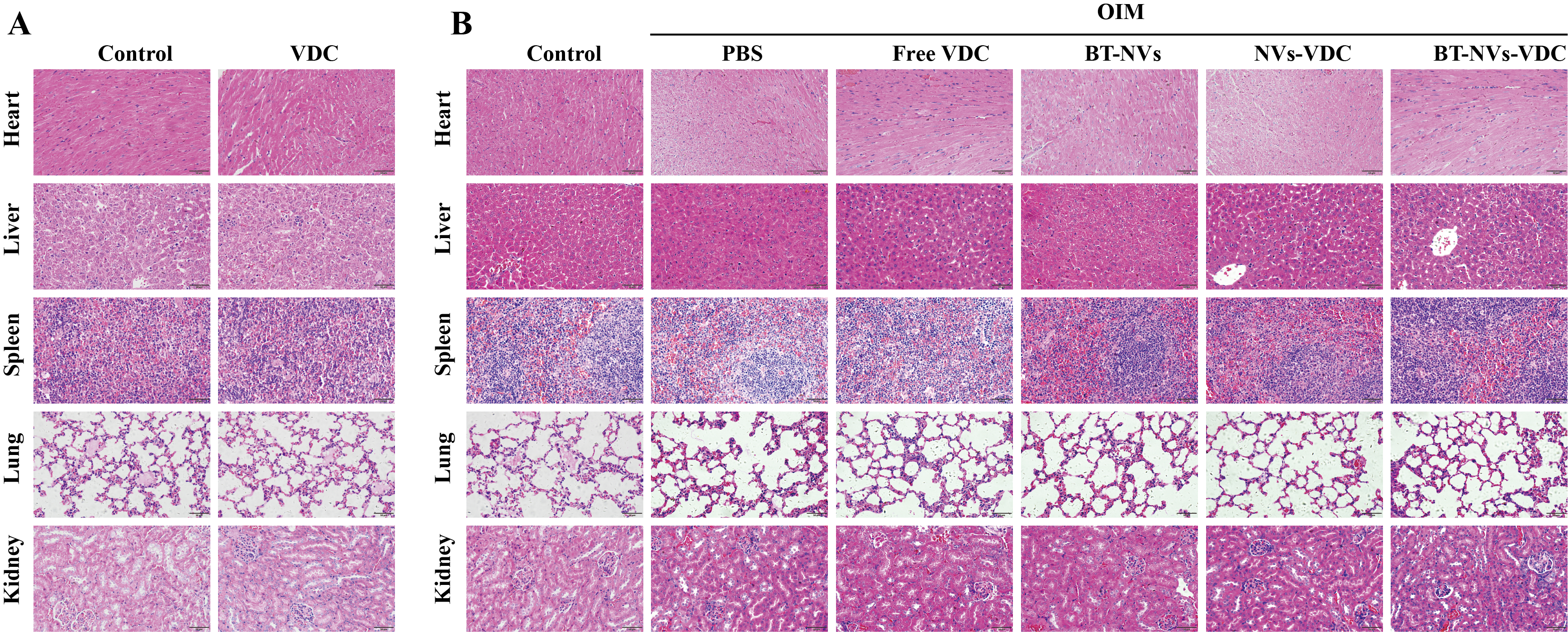


**Figure S10** Representative H&E staining images of the main organs of mice (heart, liver, spleen, lung, and kidney) from the fracture model (A) and osteogenesis imperfecta model (B). Scale bar, 50 μm (n = 5).


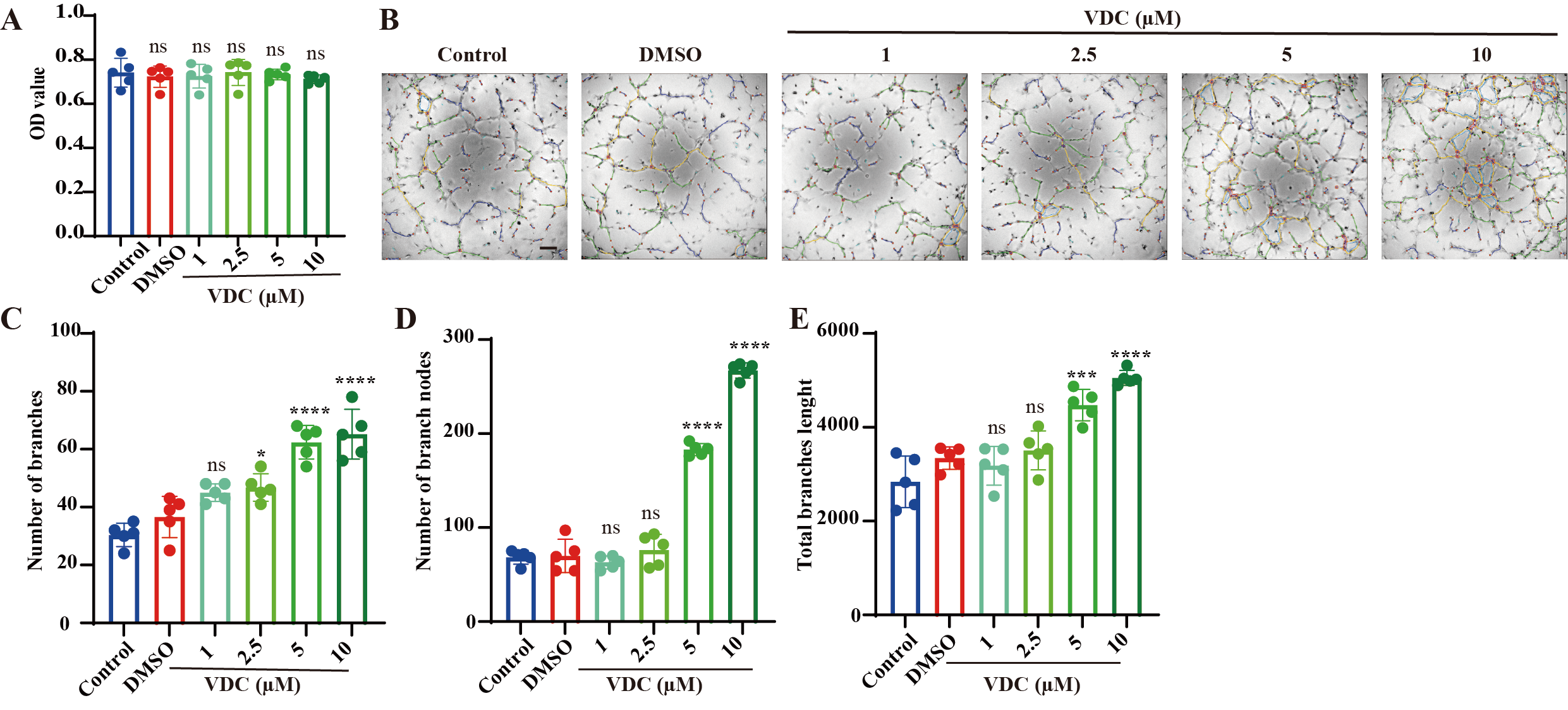


**Figure S11** VDC promotes the angiogenesis. (**A**) The cytotoxicity of VDC was assessed using the CCK-8 assay. The HUVEC cells were treated with VDC (1, 2.5, 5, 10 μM) for 48 h. Representative images (**B**) and relative quantification of tube branch numbers (**C**), tube branch nodes (**D**), tube branch length (**E**) of a Matrigel tube formation assay with HUVECs. The number of nodes (pink dots), master junctions (pink circles), master segments (yellow), meshes (light blue), branches (green), and isolated segments (blue) are shown. Scale bar: 250 μm. n = 5. Data represent mean ± SD, ns: not significant, **p* < 0.05, ***p* < 0.01, ****p* < 0.001, *****p* < 0.0001 vs the DMSO group (0.1%DMSO) by one-way ANOVA with Tukey’s post-hoc test.

**
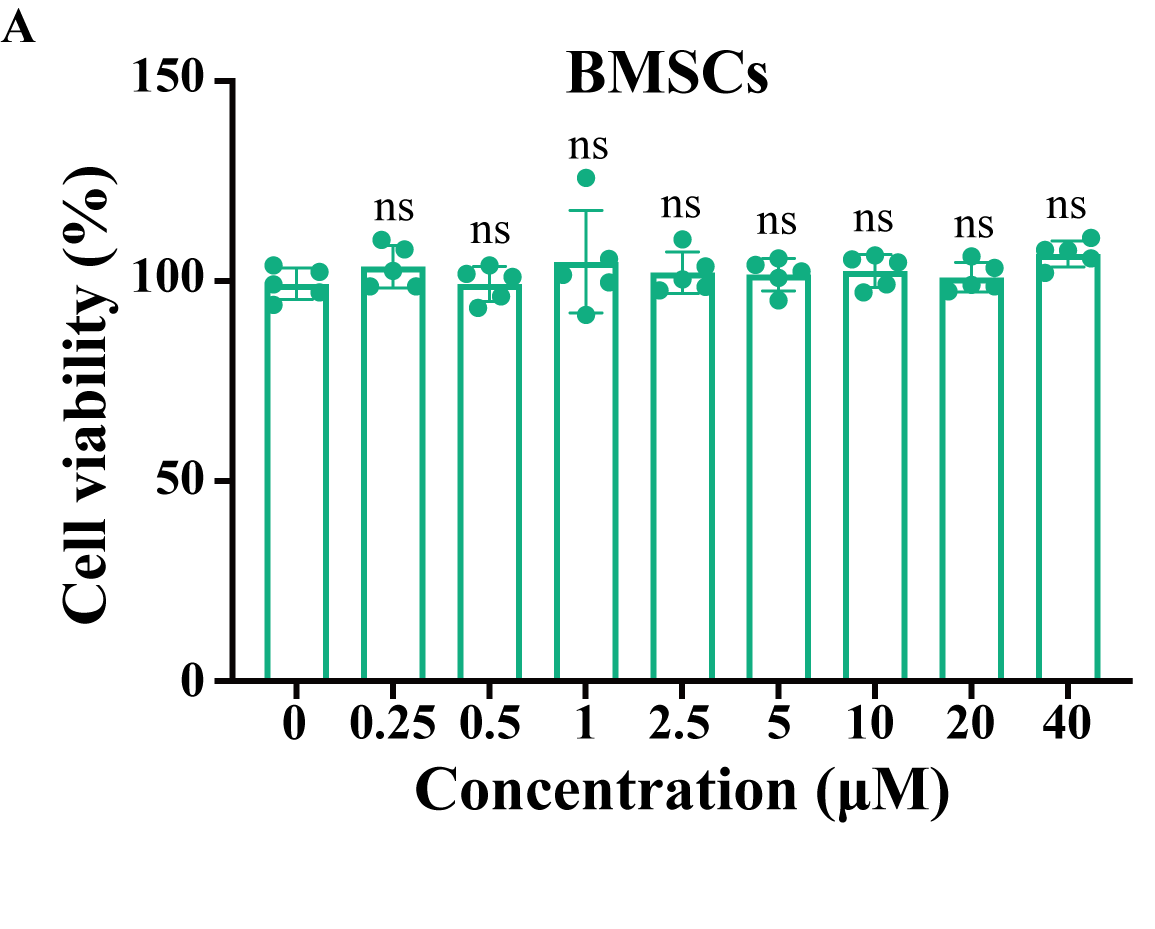
**

**Figure S12** The cytotoxicity of VDC was assessed using the CCK-8 assay. The BMSCs cells were treated with VDC (1, 0.25, 0.5, 1, 2.5, 5, 10, 20, 40 μM) for 48 h (n = 5). Data represent mean ± SD, ns: not significant vs the 0 μM concentration group (0.1% DMSO) by one-way ANOVA with Tukey’s post-hoc test.

**Table S1** Specific primers with SYBR Green (based on the mouse sequences)

| Name | Primers |
| --- | --- |
| *Hivep3* For | CTGGTTCCATCCAACTCCCGAA |
| *Hivep3* Rev | CCTCTCTTGGAAGTGGGAGTAC |
| *Alpl* For | CCAGAAAGACACCTTGACTGTGG |
| *Alpl* Rev | TCTTGTCCGTGTCGCTCACCAT |
| *Runx2* For | CCTGAACTCTGCACCAAGTCCT |
| *Runx2* Rev | TCATCTGGCTCAGATAGGAGGG |
| *Atf4* For | AACCTCATGGGTTCTCCAGCGA |
| *Atf4* Rev | CTCCAACATCCAATCTGTCCCG |
| *Sp7* For | GGCTTTTCTGCGGCAAGAGGTT |
| *Sp7* Rev | CGCTGATGTTTGCTCAAGTGGTC |
| *Ibsp* For | AATGGAGACGGCGATAGTTCCG |
| *Ibsp* Rev | GGAAAGTGTGGAGTTCTCTGCC |
| *Bglap* For | GCAATAAGGTAGTGAACAGACTCC |
| *Bglap* Rev | CCATAGATGCGTTTGTAGGCGG |
| *Hprt* For | CTGGTGAAAAGGACCTCTCGAAG |
| *Hprt* Rev | CCAGTTTCACTAATGACACAAACG |
| *Slit3* For | TCCAGTGTTCCTGAAGGCTCCT |
| *Slit2* Rev | TGGCAATGCCAGGCTCCTTGTA |

**Table S2** The names of 251 marine natural compounds (**1**-**251**)

| No | Name |
| --- | --- |
| 1 | Citrinin |
| 2 | GKK1032 |
| 3 | (Z,Z)-9,12-Ocadecadienoic acid methyl ester |
| 4 | Emodin |
| 5 | Phenol A |
| 6 | Citrinin H2 |
| 7 | Decarboxydihydrocitrinin |
| 8 | Dihydrocitrinin |
| 9 | Pinselin |
| 10 | Neocyclocitrinol C |
| 11 | 2,3-Butanediol |
| 12 | Asperbiphenyl |
| 13 | Sclerotinin C |
| 14 | (3*R**,4*S**)-6,8-Dihydroxy-3,4,7-trimethylisocoumarin |
| 15 | Dicitrinone F |
| 16 | Brevianamide Q |
| 17 | Brevianamide R |
| 18 | Sorbiallisatol B |
| 19 | Dicitrinin A |
| 20 | Diorcinol D |
| 21 | 4-(2-Hydroxy-1-methylpropyl)-3,6-dimethyl-1,2-benzenediol (ACI) |
| 22 | 3-Carboxyindole |
| 23 | 3,4-Dihydro-6,8-dihydroxy-3,4,5-trimethyl-1*H*-2-benzopyran-7-carboxylic acid |
| 24 | Sorbiallisatol A |
| 25 | Benzoic acid |
| 26 | *N*-Acetyltyramine |
| 27 | 3-Indoleglyoxamide |
| 28 | (+)-Austrosene |
| 29 | Sclerotinin B |
| 30 | Citreorosein |
| 31 | Dihydrocitrinone |
| 32 | 4-Hydroxybenzoic acid |
| 33 | *trans*-Ferulic acid |
| 34 | Citreohybriddione A |
| 35 | Aloesol |
| 36 | Monodictyphenone |
| 37 | Glulisine A |
| 38 | Prolyl-2-(1′,1′-dimethylallyl)tryptophyldiketopiperazine |
| 39 | Isocoumarin, 3,4-dihydro-6,8-dihydroxy-3,4,5,7-tetramethyl- (8CI) |
| 40 | 2-(2-Methylbut-3-en-2-yl)-1*H*-indole-3-carbaldehyde |
| 41 | Citrinal B |
| 42 | 3a,12c-Dihydro-8-hydroxy-6,11-dimethoxy-7*H*-furo[3′,2′:4,5]furo[2,3-*c*]xanthen-7-one (ACI) |
| 43 | 4,6-Dihydro-8-hydroxy-3,4,5-trimethyl-6-oxo-3*H*-2-benzopyran-7-carboxylic acid (ACI) |
| 44 | Brevianamide K |
| 45 | Sescandelin B |
| 46 | 3-[(4-Hydroxyphenyl)methyl]-6-(phenylmethyl)-2,5-piperazinedione (ACI) |
| 47 | (24*S*)-24-ethylcholesta-3β,5α,6α-triol |
| 48 | Notoamide B |
| 49 | Notoamide X |
| 50 | Speramides B |
| 51 | Taichunamide G |
| 52 | Stephaochratidin A |
| 53 | Stephacidin A |
| 54 | Notoamide R |
| 55 | Notoamide E |
| 56 | Notoamide D |
| 57 | Taichunamide F |
| 58 | Stephacidin B |
| 59 | 6-*epi*-notoamide G |
| 60 | Notoamide I |
| 61 | Sclerotiamide |
| 62 | Notoamide Q |
| 63 | Waikialoid A |
| 64 | Sclerotiamide B |
| 65 | Asperlactone |
| 66 | (3*R*,4*S*)-3,4-dihydro-4,5,8-trihydroxy-3-methyl-1H-2-benzopyran-1-one |
| 67 | 6-Hydroxyramulosin |
| 68 | 9-Chloro-8-hydroxy-8,9-deoxyaspyrone |
| 69 | Aspyrone |
| 70 | Diorcinol |
| 71 | *cis*-4-Hydroxym-ellein |
| 72 | 3b-Hydroxyergosta-8,24(28)-dien-7-one |
| 73 | 7-Nor-ergosterolide |
| 74 | Circumdatins G |
| 75 | (3*R*,4*R*)-(－)-4-hydroxymellein |
| 76 | 3-Methoxy-4-methyl-2,4-dien-pentanoic acid |
| 77 | 13-*oxo*-9*E*,11*E*-octadecadienoic acid |
| 78 | Rabdosia acids A |
| 79 | Kotanin |
| 80 | Fonsecinone D |
| 81 | Dianhydro-aurasperone C |
| 82 | Fonsecinone B |
| 83 | Fonsecinone A |
| 84 | Aurasperone A |
| 85 | Carbonarone A |
| 86 | Sohirnone A |
| 87 | Aspernigrin A |
| 88 | Eutypoid C |
| 89 | Orlandin |
| 90 | Desertorin B |
| 91 | (+)-Nigenolide B |
| 92 | (+)-Nigenolide E |
| 93 | (−)-Nigenolide E |
| 94 | (−)-Nigenolide B |
| 95 | (−)-Nigenolide C |
| 96 | (+)-Nigenolide C |
| 97 | Eutypoid B |
| 98 | Nigerasperones A |
| 99 | (−)-Nigenolide D |
| 100 | (−)-Nigenolide A |
| 101 | (+)-Nigenolide D |
| 102 | (+)-Nigenolide A |
| 103 | Nigenolide H |
| 104 | Nigenolide F |
| 105 | Eutypoid E |
| 106 | Nigenolide G |
| 107 | Pyranonigrin A |
| 108 | Helvafuranone |
| 109 | Hepialiamide B |
| 110 | Hepialiamide A |
| 111 | Hepialide |
| 112 | Hepialiamide C |
| 113 | Norcyclocitrinoicid A |
| 114 | Norcyclocitrinoicid B |
| 115 | 23-Oxoneocyclocitrinol |
| 116 | 23-O-Methylneocyclocitrinol B |
| 117 | 23-O-Methylneocyclocitrinol D |
| 118 | *(threo)*-23-*O*-Acetylneocyclocitrinol |
| 119 | *threo*-24-*O*-acetylneocyclocitrinol |
| 120 | *(erythro)-23-O-*Acetylneocyclocitrinol |
| 121 | 20*R*-24-oxocyclocitrinol |
| 122 | (20*S*)-24-oxocyclocitrinol |
| 123 | Neocyclocitrinol A |
| 124 | Neocyclocitrinols B |
| 125 | Neocyclocitrinols C |
| 126 | Neocyclocitrinols D |
| 127 | *threo*-23-*O*-methylneocyclocitrinol |
| 128 | 22-*O*-acetylisocyclocitrinol A |
| 129 | Isocyclocitrinols A |
| 130 | Isocyclocitrinols B |
| 131 | Antineocyclocitrinol A |
| 132 | Penicisteroid D |
| 133 | Penicisteroid E |
| 134 | Penicisteroid F |
| 135 | Penicisteroid G |
| 136 | Penicisteroid H |
| 137 | Penicisteroid A |
| 138 | Penicisteroid C |
| 139 | Anicequol |
| 140 | Ergosta-7,22-diene-3*β*,5*α*,6*β*,9*α*-tetraol |
| 141 | (22*E*,24*R*)-3*β*,5*α*-trihydroxy-ergost-7,22-dien-6-one |
| 142 | (3*β*,5*α*,6*β*,22*E*)-Ergosta-7,22-diene-3,5,6-triol |
| 143 | (3*β*,5*α*,6*β*,22*E*)-6-Methoxyergosta-7,22-diene-3,5-diol |
| 144 | 5*α*,6*α*,8*α*,9*α*-diepoxy-(22*E*,24*R*)-ergoxt-22-ene-3*β*,7*β*-diol |
| 145 | Ergosterol peroxide |
| 146 | Ergosterol |
| 147 | Topsentisterol D3 |
| 148 | (24*S*)-24-ethylcholesta-3*β*,5*α*-diol-6-one |
| 149 | Viridicatol |
| 150 | Penicopeptide A |
| 151 | Conidiogenones B |
| 152 | Conidiogenone D |
| 153 | Conidiogenones I |
| 154 | Conidiogenone G |
| 155 | Conidiogenones L |
| 156 | Meleagrin |
| 157 | Roquefortine C |
| 158 | Roquefortine F |
| 159 | (5*S*)-5-(1*H*-Indol-3-ylmethyl)-2,4-imidazolidione |
| 160 | Sorbicillin |
| 161 | 2',3'-Dihydrosorbicillin |
| 162 | Trichodimerol |
| 163 | Dihydrotrichodimerol |
| 164 | Citrehybridonol B |
| 165 | Andrastin G |
| 166 | Andrastone B |
| 167 | Andrastone C |
| 168 | Andrastone D |
| 169 | Andrastone E |
| 170 | Andrastone F |
| 171 | Andrastone G |
| 172 | Andrastone H |
| 173 | 3-Deacetylcitreohybridonol |
| 174 | Citreohybridonol |
| 175 | Citreohybriddione A |
| 176 | *epi*-Citreohybriddione A |
| 177 | Andrastin F |
| 178 | Meroterpenthiazole A |
| 179 | Alliisativins A |
| 180 | Alliisativins B |
| 181 | Alliisativin C |
| 182 | Alliisativin D |
| 183 | Alliisativin E |
| 184 | Alliisativin F |
| 185 | Macrophorin A |
| 186 | PenicilliuminB |
| 187 | Penicyclone A |
| 188 | (1*S*,5*S*,6*S*)-5-Hydroxy-4-(hydroxymethyl)-1-(((1*S*,8a*S*)-5,5,8a-trimethyl-2-methylenedecahydronaphthalen-1-yl)methyl)-7-oxabicyclo[4.1.0]hept-3-en-2-one |
| 189 | *N*-Formyl-*L*-tyrosine ethyl ester |
| 190 | Hydroxyphenyl)amino]-4-oxo-methyl ester butanoic acid |
| 191 | 4-[(3,5-Dihydroxy-3-methyl-1-oxopentyl)amino]butanoic acid |
| 192 | 4-[(5-Hydroxy-3-methyl-1-oxo-2-penten-1-yl)amino] butanoic acid |
| 193 | Fuscoatramide |
| 194 | (3*R*,4*R*,5*S*)-2-(((E)-2-aminovinyl)amino)-5-(hydroxymethyl)tetrahydrofuran-3,4-diol |
| 195 | Cytidine |
| 196 | 1-(5,6-Dimethyl-2-pyrazinyl)-1,2,3,4-butanetetrol |
| 197 | Nicotine acid |
| 198 | WortmannilactoneE |
| 199 | WortmannilactoneF |
| 200 | Penicyclones A |
| 201 | Penicyclones B |
| 202 | Penicyclones C |
| 203 | Pestaloficiol M |
| 204 | Pestaloficiol B |
| 205 | 5-methylbenzene-1,3-diol |
| 206 | Ergosta-4,6,8,22*E*-tetraen-11*β*-ol |
| 207 | 5*α*,6*α*-Epoxyergosta-8(14)22-dien-3*β*-ol |
| 208 | 3*β*,15*β*-Dihydroxyl-(22*E*,24*R*)-ergosta-5,8(14),22-trien-7-one |
| 209 | 3*β*,15*α*-Dihydroxyl-(22*E*,24*R*)-ergosta-5,8(14),22-trien-7-one |
| 210 | (3*β*,22*E*)-Ergosta-5,8(14),22-triene-7-one |
| 211 | 3*β*-Hydroxyl-(22*E*,24*R*)-ergosta-5,8,14,22-tetraen-7-one |
| 212 | Ergosta-4,6,8(14),22-tetraen-3-one |
| 213 | Melithasterol B |
| 214 | (22*E*)-5*α*,8*α*-epidioxyergosta-6,22-dien-3β-ol |
| 215 | (22*E*,24*S*)-5*α*,8*α*-Epidioxy-24-methyl-cholesta-6,9(11),22-trien-3β-ol |
| 216 | (22*E*,24*R*)-6*β*-methoxyergosta-7,9(11),22-triene-3β,5α-diol |
| 217 | Andrastone A |
| 218 | Incisterol A2 |
| 219 | Demethylincisterol A |
| 220 | (3*β*,22*E*)-stigmasta-5,9(11),22-trien-3-yl heptadecanoate |
| 221 | Penicillone B |
| 222 | 2,2'-Oxybis(1,4-di-tert-butylbenzene) |
| 223 | Andrastone B |
| 224 | 1,2-*Bis*(4-hydroxyphenyl)ethanone |
| 225 | CrypticinB |
| 226 | 3-hydroxy-2-methoxy-benzenebutanoic acid |
| 227 | Pyrocatechol |
| 228 | 4-Hydroxybenzaldehyde |
| 229 | 2,3-Dihydroxy-Benzoic acid |
| 230 | 2,3-Dihydroxy-benzeneacetic acid |
| 231 | Methyl-β-D-Glucopyranoside |
| 232 | Tetrahydro-3-hydroxy-3-methyl-2H-pyran-2-one |
| 233 | 4-Methyl-5,6-dihydro-2H-pyran-2-one |
| 234 | 4-(Hydroxymethyl)-5,6-dihydro-2H-pyran-2-one |
| 235 | 4-(2-Hydroxyethyl)furan-2(5H)-one |
| 236 | (*E*)-5-Hydroxy-3-methylpent-2-enoic acid |
| 237 | 9-Octadecenyl iodide |
| 238 | Stearic acid |
| 239 | Methyl linoleate |
| 240 | Linoleic acid amide |
| 241 | 9,12-Octadecadienoic acid (*Z*,*Z*)-2,3-dihydroxypropyl ester |
| 242 | n-Hexadecanoic acid methylester |
| 243 | (*Z*)-10-Eicosenoic acid methyl ester |
| 244 | Penidihydrocitrinin A |
| 245 | Penidihydrocitrinin B |
| 246 | Penidihydrocitrinin C |
| 247 | Neotricitrinols A |
| 248 | Neotricitrinols B |
| 249 | Neotricitrinols C |
| 250 | (3*R*,4*S*)-3,4,5-Trimethylisochromane-6,8-diol |
| 251 | (2*R*,3*S*)-2,3,4-Trimethyl-2,3-dihydrobenzofuran-5,7-diol |
